# Fluorescence of KCl Aqueous Solution: A Possible Spectroscopic Signature of Nucleation

**DOI:** 10.1021/acs.jpcb.2c01496

**Published:** 2022-03-28

**Authors:** Anna Maria Villa, Silvia Maria Doglia, Luca De Gioia, Antonino Natalello, Luca Bertini

**Affiliations:** Department of Biotechnology and Biosciences, University of Milano-Bicocca, Piazza della Scienza 2, 20126 Milan, Italy

## Abstract

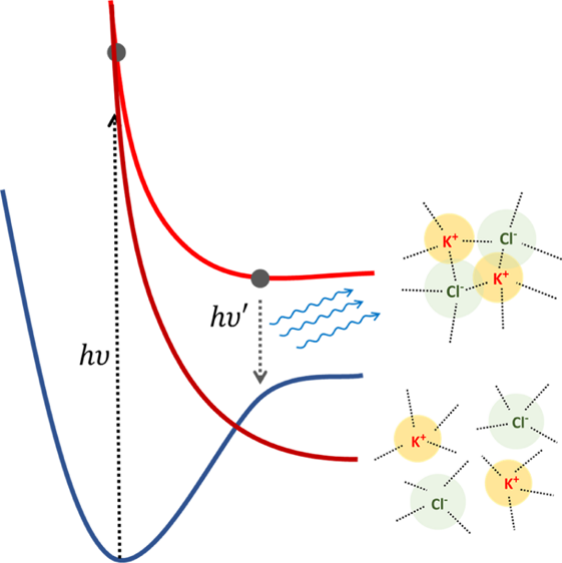

Ion pairing in water solutions alters both the water hydrogen-bond network and ion solvation, modifying the dynamics and properties of electrolyte water solutions. Here, we report an anomalous intrinsic fluorescence of KCl aqueous solution at room temperature and show that its intensity increases with the salt concentration. From the ab initio density functional theory (DFT) and time-dependent DFT modeling, we propose that the fluorescence emission could originate from the stiffening of the hydrogen bond network in the hydration shell of solvated ion-pairs that suppresses the fast nonradiative decay and allows the slower radiative channel to become a possible decay pathway. Because computations suggest that the fluorophores are the local ion-water structures present in the prenucleation phase, this band could be the signature of the incoming salt precipitation.

## Introduction

In the last few decades, the view of aqueous solutions of salts and small molecules as homogeneous at the molecular level has been challenged by an increasing number of studies. Indeed, different experimental studies have recently shown that aqueous solutions are nonuniform at the mesoscopic level and that solute-rich regions with a diameter in the range of 100–300 nm are dispersed in a bulk solution of lower concentration.^1−7^ At high concentrations, near the saturation, these solute-rich clusters have been suggested to play a fundamental role in crystal nucleation through a two-step mechanism according to which the crystalline nuclei form within these solute-rich mesoscopic clusters.^8−14^

Computational studies indicate that, in electrolyte solutions, the water molecules forming the ion hydration shell undergo a slowing down of their rotational and translational dynamics.^15−19^ By ultrafast X-ray absorption spectroscopy,^20^ a stiffening of the water hydrogen bond network in the regions around charged solute molecules has been observed, with a qualitative estimation of the dimension of these regions of about 106 nm, in agreement with the dimension of the mesoscopic solute-rich clusters observed by dynamic light scattering.^4^ In addition, terahertz spectroscopy of lactose solutions has shown a solute-induced retardation of water dynamics,^21^ and H-bonded water has been observed to give an important contribution to intrinsic fluorescence in amyloid-like fibrils.^22,23^

These findings have been supported by theoretical investigations at various levels of theory, showing that liquid water under ambient conditions presents a spontaneous transient porosity, with the formation of “voids” that tend to clusterize and can host ions, molecules,^24,25^ and even small polymers.^26^ These voids are surrounded by rings of hydrogen-bonded water molecules forming high-density patches.^24,25,27,28^

The organization of ions in electrolyte water solutions has been the subject of intensive investigations both experimentally and by computer simulations in order to elucidate the structure and dynamics of the water molecules in the immediate vicinity of the ions as well as their long range effect.^17,29−36^ Solvated ions of opposite charge can form ion pairs and clustering even at a low concentration;^17^ in particular, three types of ion pairs have been described, depending on the number of water molecules that separate the two ions: contact ion pairs (CIPs) where the cation and anion are in contact, solvent-shared ion pairs (SIPs), or solvent separated ion pairs (SSIPs) where one or two water molecules separate the two ions.

When the salt concentration increases, SSIPs can develop to SIPs and then to CIPs^17^ where different types of ion pairs play a crucial role on water dynamics and the H-bond network geometry. The different pairing states are in equilibrium at a given concentration^37^ and their reciprocal stability depends on the salt concentration and its chemical nature.^32,38^ For instance, in NaCl and KCl solutions, there is an increase in CIPs and a decrease in SSIP/SIPs upon increasing concentration,^39^ and in KCl and Ca_3_(PO_4_)_2_, CIPs are favored in prenucleation steps.^13,38^ In contrast, no evidence of CIPs is found at a moderate concentration for MgCl_2_ and LaCl_3_ or sodium acetate and Ca(ClO_4_)_2_.^32,33,38,40^ According to Chen et al.,^19^ water perturbation in electrolyte solutions appears at low ion concentrations (10 μM) and can be explained by a long-range orientation order in the H-bond network. One effect of this perturbation is the slowing down of the water molecule dynamics in the immediate vicinity of the ions.^18,19^ This slowing down could also affect the low-energy excited-state dynamics, thus reducing its nonradiative decay to the ground state.

In a previous paper,^41^ we have reported the anomalous fluorescence emission of HCl and NaOH aqueous solutions and assigned this anomalous emission to the formation, around the ionic species, of transient domains characterized by a more rigid H-bonded network with respect to that of bulk water. The stiffness of this network prevents the vibrational relaxation of the excited state, allowing fluorescence emission. In the particular case of HCl, time-dependent density functional theory (TD-DFT) computations unveiled that the emission involves the H^+^ retarded hopping to the nearest neighbor H_2_O molecule of the hydronium ion.

In this paper, we show that the aqueous solutions of KCl and NaCl present an anomalous fluorescence emission in the same wavelength range of HCl and NaOH whose intensity increases with the concentration. We suggest that, also in this case, the fluorescence emission originates from a slowing down of the vibrational/rotational dynamics of ion pairs due to the stiffening of water hydrogen bonds in the hydration shell of solvated ions and that the spectral features of this anomalous fluorescence could be related to the presence of crystal prenucleation nuclei within ion clusters in solution.

## Materials and Methods

Fluorescence emission spectra of KCl (Sigma-Aldrich, 99.0–100.5%) aqueous solutions in the concentration range between 4 M and 1.28 mM have been collected with a Cary Eclipse spectrofluorometer^42−44^ at 25 °C, scan speed of 60 nm/min. For comparison, the excitation and emission spectra of NaCl (Sigma-Aldrich, ≥99.0%) solutions are measured in the same conditions and concentration range. Absorption spectra were recorded at room temperature by a Jasco V-550 spectrophotometer in the following conditions: 1.0 nm bandwidth, 1.0 nm data pitch, and 100 nm/min scanning speed.

All the computations presented here have been carried out using DFT and TD-DFT calculations implemented in the TURBOMOLE suite^45^ on a series of KCl/water clusters that mimic the KCl solution. We adopted B-LYP^46,47^ pure functional and TZVP basis sets^48^ using resolution-of-the-identity technique^49^ to speed up calculations. Electronic spectra have been obtained first generated by TDDFT computations and then convoluted using oscillator strength weighted Gaussian distribution functions centered on the computed excitation energies (nm) with half widths at half-maxima of 70 nm. The B-LYP level of theory adopted nicely reproduces ion properties^50^ in particular for the K^+^ ion^51^ and NaCl or CaCl_2_ dissociation at the ab initio MD level.^52−55^ To validate the choice of the level of theory for the system and the properties investigated in this paper, we computed the S_1_ excitation energy for two (KCl)(H_2_O)_54_ models in their optimized geometry as a function of seven different DFT pure and hybrid functionals. All the levels of theory provide the same description of S_1_ excitation in terms of CT and mono-electronic transition. We observe that the B-LYP excitation energy is in better agreement with the experimental value as obtained from absorption spectra (vide infra and Supporting Information, Table S2).

All structures considered in this paper were selected using a classical mechanism protocol explained in the Supporting Information.

## Results and Discussion

### Fluorescence Spectra of KCl Solutions

**Figure 1:**
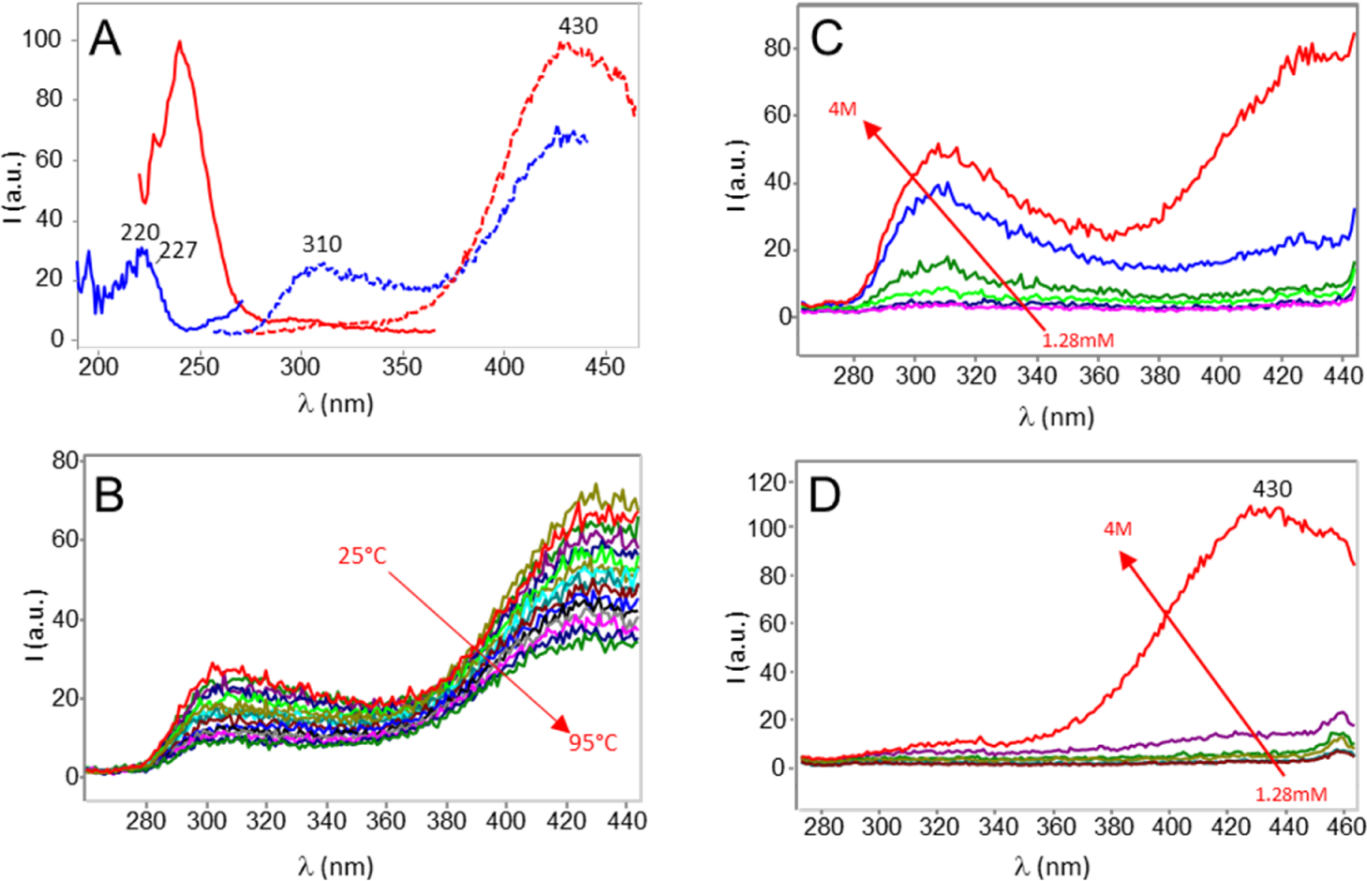
(A) Excitation and emission spectra of 4 M KCl in water. Excitation spectrum with emission centered at 310 nm (blue line) and at 430 nm (red line); emission spectrum with excitation centered at 227 nm (dotted blue line) and at 240 nm (dotted red line). (B) Fluorescence emission spectra of 4 M KCl aqueous solutions at different temperatures, from 25 to 95 °C; excitation at 227 nm. (C,D) Fluorescence emission spectra of KCl aqueous solutions at different concentrations from 1.28 mM to 4 M; (C) excitation at 227 nm, (D) excitation at 240 nm. Conditions: spectrofluorometer, Varian Cary Eclipse; photomultiplier gain, 900 V; excitation slit, 5 nm; emission slit, 5 nm.

In Figure 1A, we report the fluorescence excitation (continuous lines) and emission (dotted lines) spectra of a 4 M aqueous solution of KCl. Upon excitation at 227 nm, two bands are observed in the emission spectrum (dotted blue line): a weaker emission peaked at 310 nm and a stronger emission peaked at 430 nm. The excitation spectrum (blue line), collected with emission centered at 310 nm, presents a peak and a shoulder at 220 and 227 nm, respectively. The excitation spectrum with emission centered at 430 nm (red line) shows a strong band at 240 nm, with a shoulder at 227 nm. By exciting at 240 nm, the emission spectrum (dotted red line) shows a single band with a maximum at 430 nm. The comparison between the emission spectra in Figure 1A suggests the presence in solution of two fluorescent structures: one with emission at 310 nm and the other with emission at 430 nm.

The presence, upon excitation at 227 nm, of both emissions at 310 and 430 nm is due to the superposition of the excitation bands of the two fluorescent structures, as indicated by the Gaussian analysis of the excitation spectrum collected with an emission at 430 nm (Figure S1). The analysis shows that there is an overlap between the tail of the large Gaussian band peaked at 240 nm and the 227 nm band. Therefore, upon excitation at 227 nm, both fluorescent structures are excited, giving the observed two bands in the emission spectrum.

We determined a relative quantum yield (QY) (see Supporting Information, Figures S2–S4) of 0.0023 ± 0.0006 for the emission band at 310 nm with excitation at 227 nm, and of 0.0084 ± 0.0010 for the emission band at 430 nm with excitation at 240 nm.

To further explore the characteristics of this anomalous emission, we performed the fluorescence spectra of KCl aqueous solutions at different concentrations in the range 1.28 mM–4 M (Figure 1C,D). As can be seen, the fluorescence intensity increases with KCl concentration, indicating that this emission is due to the presence of the solute. The absorption spectra of the KCl solution confirm the presence, in the region 220–300 nm, of absorption bands at the same wavelengths of the fluorescence excitation maxima (see Figures S2 and S3).

As can be seen in Figure 1C,D, while the emission intensity of the 310 nm band increases gradually with concentration, the 430 nm band shows an abrupt intensity growth between 0.16 and 4 M, when excited either at 227 nm (Figure 1C) or at 240 nm (Figure 1D).

As reported in the literature,^17,29,32,33,36,56^ due to ion pairing, different types of ion-water structures with different ion solvations can occur in salt aqueous solutions: at a very low concentration, only hydrated free ions are supposed to be present, but at high salt concentration, CIPs also appear, with a different solvation structure.^57,58^ In this perspective, the important intensity increase of the 430 nm band at concentrations higher than 0.16 M suggests that this emission band could be attributed to those less hydrated ion/water clusters involved in the prenucleation step in which CIP formation and ion–ion interactions are crucial. In contrast, the steep growth of the 310 nm band intensity at low concentrations, followed by a moderate intensity increase for *c* > 0.8 M (Figure S5), suggests that the origin of this emission could be due to ion/water clusters in which SIP and SSIP formation is important. Finally, at very low concentrations, fully hydrated ion pairs likely prevail, that seem not able to show emission properties. Our data suggest that the stiffening of the water environment around ions and ion pairs hinders not only the vibrational relaxation on the ground-state potential energy surface (PES)^29,33,36^ but also the vibrational relaxation on the low-energy excited-state PES, thus favoring fluorescence emission.

To assess the involvement of the water environment in the observed anomalous fluorescence, the emission of KCl was measured in deuterated water (D_2_O). Noteworthy, our data show that the fluorescence intensity of KCl in D_2_O is enhanced compared to H_2_O (Figure S6). This enhancement points to solvent involvement as the necessary condition for fluorescence emission of KCl. Indeed, a kinetic isotope effect on fluorescence is expected for chromophores which participate in proton mobility processes and has been proposed to be due to the slower rate of the excited-state proton transfer in deuterated water.^59^

In order to compare our results on KCl solutions with those of another monovalent salt, we measured the fluorescence emission spectra of NaCl aqueous solutions at concentrations in the range 1.28 mM–6.16 M (Fig.S6). When excited at 227 nm, the emission spectra of NaCl solutions show a prominent band at 300 nm, similar to the 310 nm band of KCl, and an emission band at 420 nm which, in contrast to KCl solutions, displays a low intensity even at concentrations as high as 6.16 M (saturation). The emission intensity of the 420 nm band remains low also when excited at 240 nm (data not shown). These data suggest that in the NaCl solution, the formation of CIPs is reduced with respect to KCl. These differences in CIP formation between KCl and NaCl solutions are in agreement with the “law of matching water affinity” of Collins^17,29,32,60^ stating that ions with a similar size have the tendency to form CIPs, while ions with a different size prefer to stay separate in solution. Indeed, the K^+^ and Cl^–^ ions have very similar radii, while the radius of the Na^+^ ion is much smaller.^61^ Another aspect that affects the formation of CIPs is the salt hygroscopicity: the less hygroscopic salts tend to form CIPs more easily.^17^ In our case, the deliquescence relative humidity, a parameter that is higher for less hygroscopic salts, has a value of 85% for KCl and 75% for NaCl, supporting our findings that KCl has more propensity than NaCl to form CIPs. It should be noticed that ion pairing affects the structure and dynamics of the water molecules located in the electric field between the positive and negative ions, reducing the orientational freedom of these bridging waters and slowing down their motion with respect to bulk waters.^29^ This structural stiffening reduces the vibrational relaxation, preventing nonradiative decay and favoring fluorescence.

### Temperature Dependence of the Fluorescence Emission Spectra

The effect of temperature on the fluorescence intensity of the KCl solution was investigated by collecting the fluorescence emission spectra every 5 °C from 25 to 95 °C (Figure 1B). As expected, both fluorescence peaks decrease with the increase in temperature, and when we cool back the solution to 25 °C, fluorescence returns to the original intensity, indicating a reversible process. The area of both fluorescence bands decreases linearly with temperature (Figure S7), but the slopes of the interpolating lines are −15 for the 307 nm and −42 for the 430 nm band, respectively, indicating that the emission at 430 nm decreases more steeply than that at 307 nm. These results further support the hypothesis that the two emission bands are due to different fluorescent structures present in KCl aqueous solutions.

From these spectra, it is also possible to estimate, for each emission, the activation energy of the nonradiative processes that compete with fluorescence.^17,62−64^ We found that the Arrhenius plots of the natural logarithm of the emission band area versus 1/*T* are linear for both emission bands (data not shown); however, we obtained two different values of activation energy: Δ*E*_1_ = 12.45 ± 1.88 kJ/mol for the 310 nm band and Δ*E*_2_ = 7.54 ± 0.20 kJ/mol for the 430 nm band (Figure S5, Supporting Information). In a previous paper,^41^ we have shown that the fluorescence emission of 3 M HCl aqueous solution presents a single band at 300 nm (exc = 220 nm). From the Arrhenius plot of this band (data not shown), a Δ*E* of 12.5 kJ/mol can be calculated.

Interestingly, the very similar activation energies obtained for the 310 nm band of the 4 M KCl solution and for the 300 nm band of the 3 M HCl solution are in good agreement with the activation energy values found in the literature. Indeed, delayed luminescence measurements of salt solutions give a value of 12.7 kJ/mol for the activation energy required to interconvert high-density and low-density water structures.^65^ Moreover, the calculated energy barrier between strong and weak hydrogen bonds in water was reported to be of 12–13 kJ/mol^62,63^ and, by molecular dynamics simulation, a value of 13.8 kJ/mol was found for the cleavage energy of the strongest H-bonding solvating the hydronium ion.^66^ Intriguingly, all these energy values correspond to infrared frequencies in the region of water librations (about 1040 cm^–1^ for 12.45 kJ/mol and 630 cm^–1^ for 7.54 kJ/mol). In particular, for the HCl solution, a broad absorption around 1200 cm^–1^ (14.3 kJ/mol) is observed in the FTIR difference spectra,^41^ which has been attributed to the proton shuttling between two vibrationally coupled water molecules.^67^

It seems therefore possible to hypothesize that 12.45 and 7.54 kJ/mol could correspond to the energy needed to disrupt the rigid H-bond configurations responsible for the two anomalous fluorescence emission observed in our solutions, also suggesting that the two fluorescence emission bands correspond to structures with different H-bond energies. In this perspective, these findings seem to suggest that particular water libration modes are involved in the vibrational relaxation that leads to nonradiative deexcitation of our anomalous fluorophores.

### DFT and TD-DFT Modeling

To elucidate the possible mechanism of the emission process, we performed DFT and TD-DFT calculations on KCl/water clusters which are simplified models of the KCl solution at a given concentration.

The essential points of this modeling are as follows:

1. The search for a minimum energy geometry of KCl/water clusters with different ion/solvent rates and the analysis of their frontier molecular orbitals (FMOs);
2. The computation of the TD-DFT electronic absorption spectra to characterize S_1_ excitation in terms of atom-to-atom charge-transfer (CT);
3. The identification of a plausible chemical structure for the emitting fluorophore.

### General Features of the KCl/Water Cluster Electronic Structure

Before commenting on the obtained results, we underline that the search for the KCl/water cluster minimum geometry is not intended to be exhaustive of the dynamical behavior of the real system, but allows us to identify those ion/water dispositions that could be fluorescent active.

**Figure 2:**
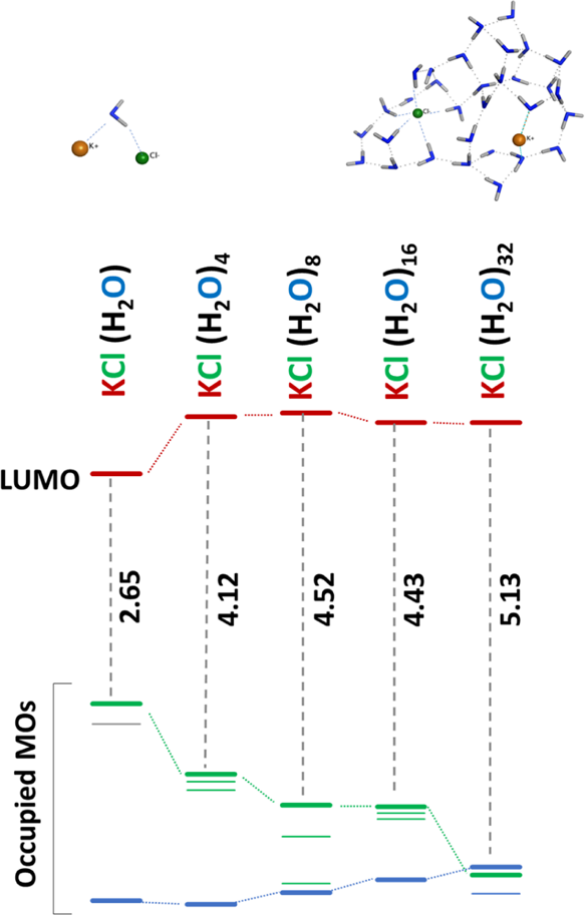
Molecular orbital energy diagram for (KCl) (H_2_O)_*n*_ clusters with the decrease in KCl/water ratio (*n* = 1, 4, 8, 16, and 32). The HOMO/LUMO gap is reported in eV, and the horizontal bars represent the values of the calculated MO energy eigenvalues. Different bar colors have been used to represent the atomic localization of HOMO and LUMO as a function of KCl/water ratio. Green: HOMO localized on Cl^–^ 3p; blue: HOMO localized on O^–^ 2p; and red: LUMO localized on K^+^ 3s. The structures reported at the top of the panel are the n = 1 and *n* = 32 minimum forms identified in this investigation. As can be seen in the figure, for low *n*, corresponding to concentrated solutions, HOMO is localized on the Cl^–^ 3p anion (green bars). Going from lower to higher *n*, that is, diluting the solution, the energy of the MO localized on Cl^–^ decreases and its stabilization increases. When *n* = 32, the HOMO corresponds to the oxygen 2p MO (blue bar). In contrast, the LUMO is localized on potassium 3s (red bars) for all the values of *n*.

The first result of this modeling is that the nature of the S_1_ excitation changes as a function of the KCl concentration: from O → K CT type at a low concentration to the Cl → K CT type at a higher concentration. We identified the changing nature of CT in our systems by investigating the electronic structure of a series of small (KCl)(H_2_O)_*n*_ clusters with *n* = 1, 4, 8, 16, and 32 (Figure 2). The mono-electronic transition in S_1_ excitation is always HOMO → LUMO, and the orbital composition of these two MOs allows us to identify the nature of S_1_ in terms of CT. While the LUMO is always localized on potassium with prevalent 4 s orbital contributions, the HOMO atomic orbital contributions depend on the chloride anion coordination number. In particular, we found two different situations: (i) when the Cl^–^–H electrostatic interactions are 4 or more, these interactions stabilize the Cl^–^ 3p lone-pair atomic orbitals and lower their energy, resulting in a HOMO localized on the 2p oxygen orbitals; (ii) when the Cl^–^–H interactions are less than 4, the Cl^–^ 3p lone-pair atomic orbitals are less stabilized, resulting in a Cl localized HOMO.

These observations suggest that for higher KCl concentration in solution, that is, for lower values of *n*, the orbital composition of HOMO changes from O 2p to Cl 3p MO, and consequently, the nature of the S_1_ excited state changes from O → K to Cl → K charge transfer type. In the real KCl solutions, low KCl/H_2_O ratios correspond to oversaturated solutions, which are unstable and have the natural tendency to show KCl precipitation. However, as recently observed by the classical MD simulation,^13^ these structures are also locally present in the prenucleation phase, that is, when the KCl concentration is near the saturation limit (4.56 M at 20 °C).

### Characterization of 1 and 2 M KCl Solution Models

To validate the observations reported above and to attempt to identify the anomalous fluorophore, we consider more realistic models, that is, (KCl)(H_2_O)_54_ (Table S1) and (KCl)_2_(H_2_O)_108_ (Table S6) 1 M and (KCl)_2_(H_2_O)_53_ (Table S5) 2 M KCl model clusters. In this validation approach, we will only consider the structures in which both K^+^ and Cl^–^ ions are buried in the solvent, as already adopted in the case of HCl/water model clusters.^41^ Although these structures could be higher in energy compared with those in which the charged ions are placed at the border of the cluster, they are clearly a better model for KCl bulk solution.

**Figure 3:**
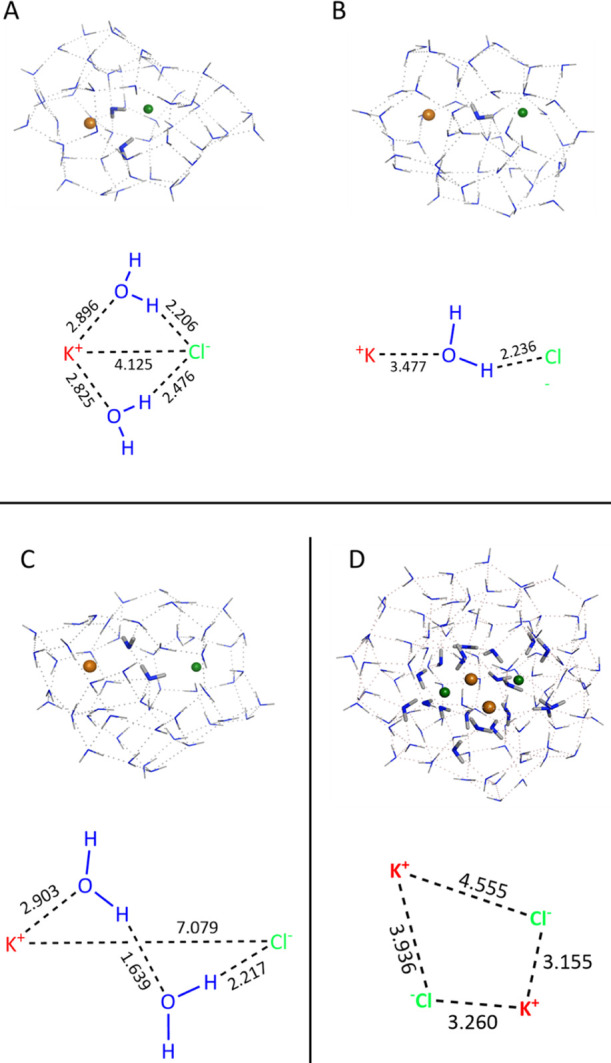
KCl(H_2_O)_54_ and (KCl)_2_(H_2_O)_106_ buried ion models. (A–C) KCl(H_2_O)_54_ models simulating CIP, SSIP, and SIP conformations. (D) (KCl)_2_(H_2_O)_106_ model that mimics a prenucleation cluster structure. Ball atoms are ions. Thicker stick models are used to put in evidence water molecules that are involved in the ion-pair interactions. Distance in Å.

In (KCl)(H_2_O)_54_ models, the K^+^ ion is on average pentacoordinated with an average K–O distance of 3.09 ± 0.21 Å, while Cl^–^ is six-coordinated with an average Cl–H distance of 2.31 ± 0.21 Å (experimental values 2.90 and 2.35 Å, respectively^68^). The average K–Cl distance is 5.56 ± 1.83 Å. In Figure 3A–C, the most stable structures are sketched mimicking solvent contact, SIP, and SSIP forms (models 1, 5, and 6 from Table S1 in the Supporting Information), where structure A results the lowest in energy among those considered here. For (KCl)_2_(H_2_O)_53_ and (KCl)_2_(H_2_O)_108_ models, the structures considered were selected with the purpose of being representative of the different arrangements of ion pairs (see details in Tables S5 and S6).

For each model, we computed the absorption spectrum evaluating the S_1_ excitation energy [262.5 ± 16.9, 274.6 ± 17.0, and 282.7 ± 18.0 nm for (KCl)(H_2_O)_54_ models, (KCl)_2_(H_2_O)_53_ and (KCl)_2_(H_2_O)_108_ models, respectively].

Comparing the experimental and TD-DFT absorption spectra (see Supporting Information, Figures S10 and S11 for details), we observe that the excitation wavelengths which include those used to collect the fluorescence emission spectra (227 and 240 nm) are due to the CT from 2p/3p orbitals FMO of oxygen and/or of chloride atoms to the potassium and/or hydrogen atoms. The weak shoulder measured at 260–270 nm for 4 M KCl (Figure S2 in the Supporting Information) can be tentatively assigned to the computed weak shoulder around 230–235 nm, which corresponds to the S_6_ excitation. The computed value of S_1_ excitation energy of 255–265 nm allows us to estimate its experimental value around 290–300 nm, which is close to the average computed values reported above. Using the same approach for (KCl)_2_(H_2_O)_108_, we obtain a value of 305 nm (Figure S13).

According to HOMO and LUMO populations, the S_1_ singlet excited state in 1 M models has often an O → K,H CT character, in which on average 0.5 electrons are transferred to K^+^, thus inducing a transient decrease of its positive atomic charge (see Supporting Information, Table S3). For 2 M KCl models, we observe a larger number of structures in which S_1_ is characterized by a Cl → K CT, confirming the results presented in Figure 2.

### Identification of a Plausible Chemical Structure for the Emitting Fluorophore

S_1_ dynamics explored within TD-DFT geometry optimization evidences that the K^+^ ion, which at the beginning is hosted in a cage made on average by 7–8 water molecules plus, in some cases, the Cl^–^ ion, moves until it reaches the structure at the crossing with the ground-state PES, thus leading to a pure radiationless process. However, in this model, the radiationless decay is mainly due to the unphysical vibrational relaxation of the water molecules at the cluster surface because the oxygen 2p localized HOMO always involves an atom that belongs to the cluster surface, as already observed in similar computations on HCl/water cluster models.^41^ To prevent this unphysical nonradiative S_1_ decay that involves the cluster surface to bring the system at the configuration of the S_1_/S_0_ PES crossing, we introduced some constraints to fix the positions of the atoms surrounding the K^+^ ion during the geometry optimization, which corresponds to make the cage around K^+^ ion more rigid, thus limiting the K^+^ motion.

We explored the S_1_ dynamics by TDDFT constrained geometry optimization. In each case, we compute Δλ (in nm, Tables S4 and S6). Because we are observing only the S_1_ ion dynamics, larger Δλ corresponds to higher ion mobility. This dynamics is induced by the CT that attenuates the ion–ion and ion-dipole electrostatic interaction. Computed Δλ values depend on a variety of factors but we observe that a higher K^+^ ion coordination number implies lower ion mobility. This implies that S_1_ ion mobility can be largely hindered by

1. Stiffening the solvent cavity that hosts the ions;
2. K^+^–Cl^–^ ion–ion interactions in SIP or CIP structures.

This stiffening induces a slowing down of the vibrational cooling of the S_1_ state and allows fluorescence emission.

This last finding led us to investigate the model clusters with a higher KCl/water ratio, that is, the (KCl)_4_(H_2_O)_106_ 2 M KCl models, focusing on those structures in which the eight ions are close to each other mimicking a small prenucleation aggregate (Figure S14 and S15 in the Supporting Information). As suggested by Fetinov et al.,^68^ these salt clusters present bulk-like properties and are stabilized by the interaction with the solvent. All S_1_ geometry optimizations converge to local minima and those characterized by low Δλ have an almost cubic unit at the center of the cluster, which suggests that ion–ion interactions on the S_1_ PES are attenuated compared to the ground state but not enough to reduce ion mobility and to slower radiationless excited-state decay.

## Discussion

On the basis of the experimental spectra and of the DFT/TD-DFT modeling, we now attempt to account for the emission properties of the KCl solution. Because pure water has no detectable emission (Figure S9 in the Supporting Information), the fluorescence of KCl solution actually depends on the presence of the solute and/or on the effect of the solute on water. In this perspective, upon excitation of the KCl solution, the emitting fluorophores will be those local structures in which the ions are involved in S_1_ mono-electronic excitation and the water H-bond network is sufficiently rigid to hinder the radiationless decay.

We observe that the TD-DFT computed absorption spectra obtained using the KCl solution models, show that the low-energy electronic excited states populated during the excitation have O → K or Cl → K CT character, depending on the KCl concentration and on the chloride ion solvation. According to mono-electronic transitions, an electron density transfer to potassium transiently decreases its positive charge, thus weakening its ion–ion and ion–dipole electrostatic network interactions. The same is true for the chloride ion and the water molecules when involved in S_1_.

Through constrained TD-DFT S_1_ geometry optimization, we attempt to characterize the dynamics of the ions in solution along the S_1_ vibrational cooling coordinate.

**Figure 4:**
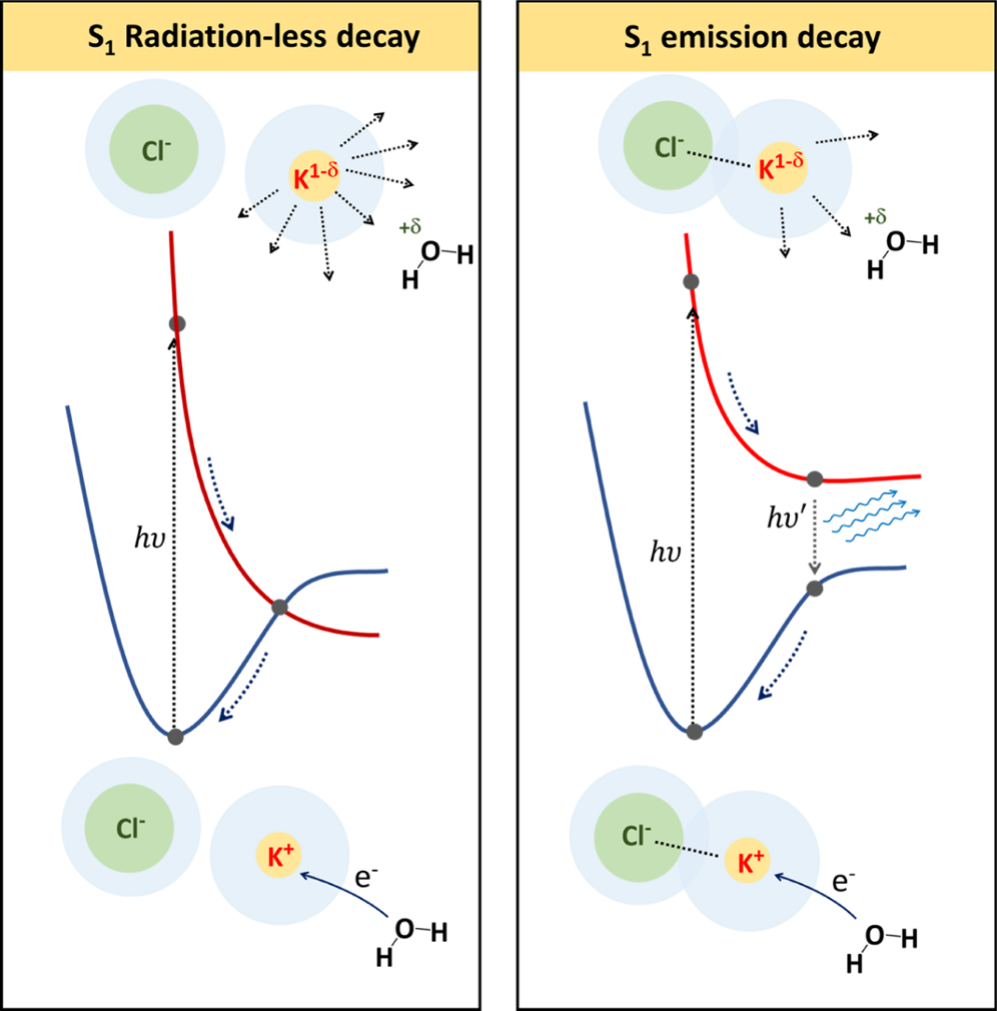
Proposed model for S_1_ ion dynamics of KCl solution at low concentration (left) and high concentration (right). At a low concentration (left), where separated hydrated free ions are the most populated ion structural arrangements, S_1_ excitation dynamics is not hindered by electrostatic interaction and allows S_1_ nonradiative decay; at a higher concentration (right), solvent shared ions or CIP structures are more frequent. In this case, S_1_ excitation dynamics is strongly hindered by ion pairing electrostatic interaction and allows emissive S_1_ decay.

In Figure 4, the hypothesis for the S_1_ excited-state dynamics is sketched at low and high KCl concentrations. In the low concentration situation, most ions involved in the S_1_ excited state are present as free hydrated ions and SSIP. The emission intensity is small, indicating that S_1_ decays mostly via radiationless mechanisms.

Upon excitation, the CT toward K^+^ ion induces the vibrational relaxation of the species involved in the CT and of their immediate solvation shells. Either in the case of O → K or Cl → K CT, at a low concentration, the structure of free hydrated ions and SSIP species is not rigid enough to prevent nonradiative decay as in pure water. In the case of KCl solutions at higher concentration, where ion pairs will be gradually more populated in the order SSIP → SIP → CIP, as already reported in the literature, ion pairs are able to slow down the vibrational and rotational dynamics of the solvent due to the medium/long range effect of their charges. These results are in good agreement, among many, with the findings of Shukla et al.^69^ that indicate a reduction in bulk water reorganization rate of approximately 30% for a 0.1–0.25 M disaccharide solution and with Stirnemann et al.^15^ that observe by classical mechanics computations that the solutes alter the dynamics of water.

In CIPs, the vibrational stiffening will originate from the short-range effect of the ions close to the species involved in the CT and the same effect will be active on the S_1_ PES.^29,70^ A similar effect would be active in the S_1_ dynamics of those structures that are present in the prenucleation phase,^13,71,72^ when the concentration is close to saturation and ion–ion interactions are strong again, although the charge transfer weakens the ion–ion and ion/dipole interactions facilitating ion mobility in solution, the slowing down of the S_1_ dynamics is still sufficient to prevent the nonradiative excited-state decay process.

The presence of two different emission bands, at 310 and 430 nm, for KCl and of one single band at 300 nm for NaCl suggests a different S_1_ dynamics when K^+^ or Na^+^ are involved in the CT, although the effect of the solutes could be not ion-specific, explaining why all concentrated solutions slow down water dynamics.^15^

This is also supported by the different values of the energy (7.54 kJ/mol for 430 nm emission; 12.45 kJ/mol for 310 nm emission) needed to disrupt the H-bond configurations responsible for the anomalous fluorescence of KCl. We tentatively assign CIP emission to the 430 nm band and SIP/SSIP to the 310 nm band. According to this hypothesis, the fact that for NaCl only the band at 300 nm is observed suggests that for this salt, the vibrational relaxation of S_1_ CIPs leads to nonradiative deexcitation of our anomalous fluorophores.

## Conclusions

To summarize, in this paper, we have reported for the first time an anomalous fluorescence emission of KCl and NaCl aqueous solutions at room temperature in the 0.00128–4 and 0.00128–6.16 M concentration range, respectively. For KCl, two emission bands at 310 nm (QY = 0.0023 ± 0.0006) and 430 nm (QY = 0.0084 ± 0.0010) are observed, with excitation at 227 and 240 nm, respectively. Only one emission band at 300 nm (QY = 0.006 ± 0.0008) was observed for the NaCl solution. The emission band intensity versus concentration and the Arrhenius plot of KCl solutions clearly suggest that the 310 and 430 nm bands are due to the emission of two different species with different activation energies for the nonradiative processes that compete with fluorescence.

Our data, together with TD-DFT modeling, suggest that the unexpected fluorescence observed in KCl and NaCl aqueous solutions could arise from the stiffening of water/ions network structures. These structures can be described as a sort of clusters, formed by ions and by their hydration spheres, with a composition depending on the concentration of the solution.

For KCl at a low concentration, where free hydrated ions and SSIP species are more populated, the S_1_ O → K or Cl → K CT excited state evolves along the vibrational cooling coordinate that rapidly brings the geometry of the system at the crossing with the ground state PES, thus resulting in a nonradiative excited-state decay process. By increasing the KCl concentration, the ion/water clusters will change in composition and become SIPs and CIPs or the more tight aggregates responsible for the nucleation process. For these types of aggregates, the nonradiative S_1_ decay mechanism is slowed down by the increased stiffness of the coordination environment of the CT active species due to either the long-range effect of water dynamics or the ion–ion interactions, which favors the emission pathway. If this is the case, at the saturation, the 430 nm fluorescence band could be the signature of the incoming salt precipitation.

Both fluorescence emission spectra and TD-DFT modeling suggest that ions in solution can form anomalous fluorophores and disclose the possible effect of ions on the dynamics of KCl and NaCl water solutions at different concentrations. We finally underline that the model proposed here is a first attempt to account for this anomalous fluorescence and does not exclude that the active mechanism for radiative decay could be the stiffening effect of ions on the water hydrogen bond network that changes the S_1_ dynamics. Indeed, as reported by Stephens et al.,^73^ molecular structures with short hydrogen bonds are able to significantly decrease the probabilities of nonradiative transition.

This intriguing property is currently prompting us to a wider investigation on the emissive properties of water solutions of solutes with different chemical natures beyond strong electrolytes.

## Supporting Information Available

Details regarding the experimental part; Gaussian analysis of the spectra; determination of the fluorescence quantum yields; calculation of the activation barrier of radiationless deactivation; absorption spectra for KCl and NaCl versus concentration; NaCl 4 M emission spectra versus temperature; emission spectra of KCl 4 M versus MillQ water; fluorescence emission spectra of 2 M KCl in H_2_O and in D_2_O; detail of DFT speciations of model KCl 1 M and 2 M solutions; benchmark of KCl(H_2_O)_54_ buried ion isomer properties versus DFT functional; TDDFT KCl absorption band assignments; and Cartesian coordinates of the main structures identified (PDF).
