# Supplementary material for: Fluorescence of KCl Aqueous Solution: A Possible Spectroscopic Signature of Nucleation
Source: J Phys Chem B. 2022 Mar 28;126(13):2564–72. doi: 10.1021/acs.jpcb.2c01496 (PMC8996234; doi:10.1021/acs.jpcb.2c01496)
Supplement: Supplementary file 1 — jp2c01496_si_001.pdf [file jp2c01496_si_001.pdf]

Supporting Information.

Fluorescence of KCl Aqueous Solution. A Possibile Spectroscopic Signature of Nucleation

Anna Maria Villa, Silvia Maria Doglia, Luca De Gioia, Antonino Natalello, Luca Bertini*
Department of Biotechnology and Biosciences, University of Milano-Bicocca, Piazza della Scienza 2, 20126 Milan, Italy.
E-mail: luca.bertini@unimib.it

1. Gaussian analysis.

A gaussian analysis was performed using the ABcurvefit application of the GRAMS/AI 8.0 (Thermo) program on the fluorescence excitation spectrum of KCl 4M. Two gaussian curves with maxima at 227 nm and 430 nm were sufficient to reproduce with good accuracy the shape of the 240 nm band.

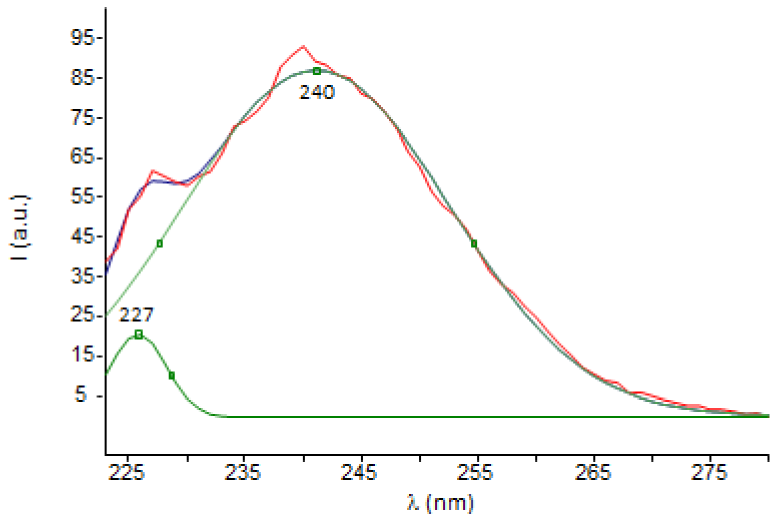

FIGURE S1. Gaussian analysis of the fluorescence emission spectrum of KCl 4M solution (exc = 430 nm). Red: original trace; green: gaussian curves; blue: fitted trace.

3. Determination of relative fluorescence quantum yield.

Absorption spectra of KCl and NaCl were collected to estimate the molar absorption coefficients for the 227 nm and 240 nm bands of KCl aqueous solution and for the 227 nm band of NaCl aqueous solution. The value of the absorption coefficient for both solutions are of about 10^{-2} cm^{-1} mol^{-1}, 6 orders of magnitude lower than that of fluorescein.[1] We determined the relative quantum yields (QY) of KCl and NaCl solutions by a comparative method using a reference sample with

known QY.[2] Phenylalanine (QY=0.024)[3] was employed as reference fluorophore since its absorption (λ_{max}= 257 nm) and emission (λ_{em}= 280 nm) are in the same spectral region of the solutions to test. The absorption and emission spectra of phenylalanine, KCl and NaCl solutions at different concentrations were collected in the same instrumental conditions. For each solution the concentration range was carefully chosen in order to keep the absorbance at the excitation wavelength lower than 0.1.[2,4] For the KCl solution we obtained a QY of about 0.0023 (SD=0.0006) for the emission band at 310 nm (exc = 227 nm), and of 0.0084 (SD=0.0010) for the emission band at 430 nm (exc = 240 nm). For the NaCl solution, a QY of 0.006 (S.D.=0.0008) was obtained (exc = 227 nm; em = 300 nm).

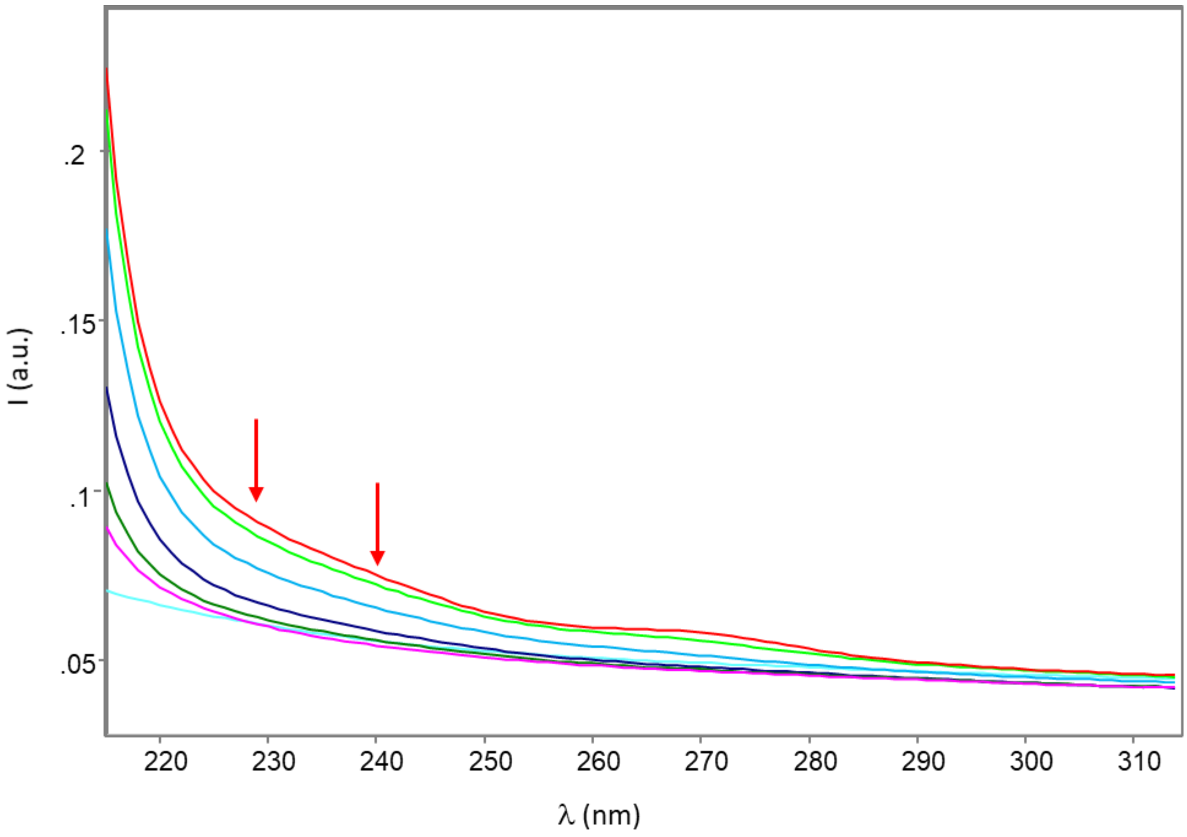

Figure S2. Absorption spectra of H_2O (light blue) and of KCl aqueous solutions at different concentrations from 0.25M to 4M. The red arrows indicate the excitation wavelengths used in fluorescence measurements.

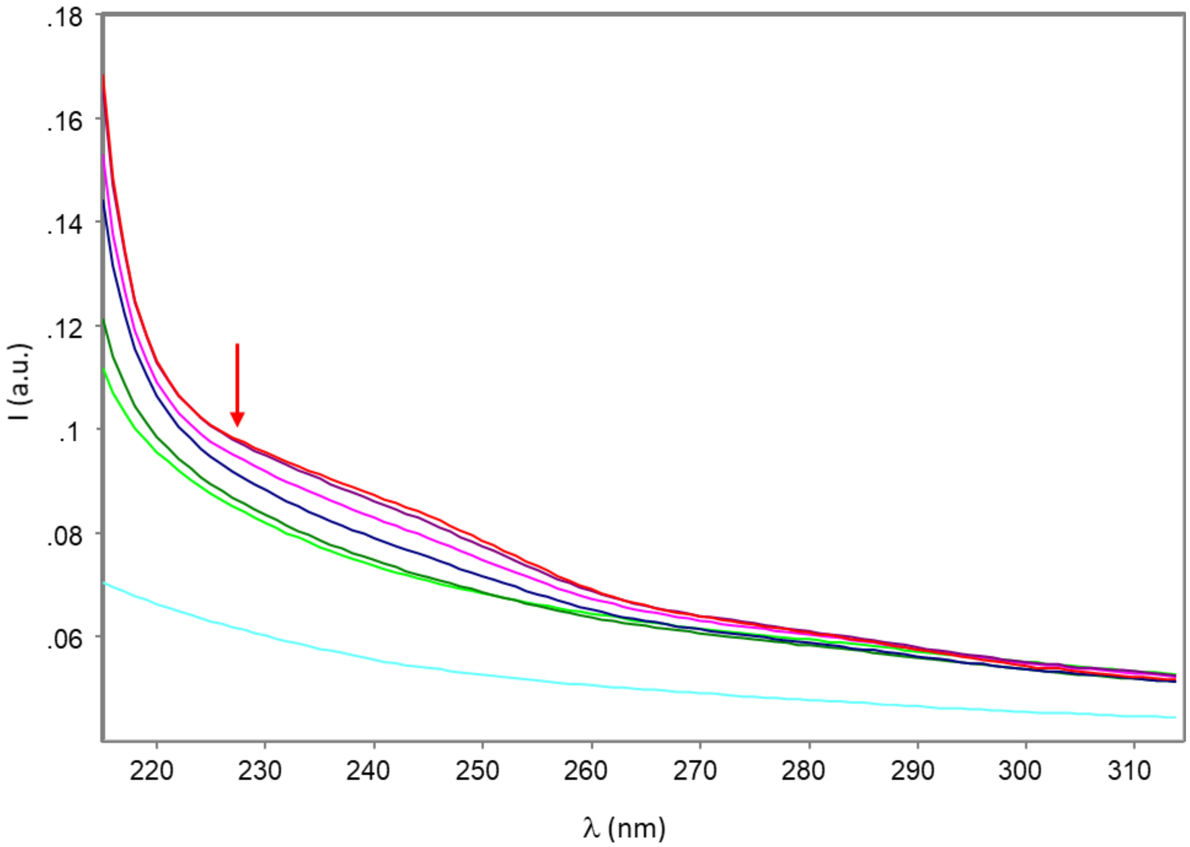

Figure S3. Absorption spectra of H_2O (light blue) and of NaCl aqueous solutions at different concentrations from 0.5M to 5M. The red arrow indicates the excitation wavelength used in fluorescence measurements.

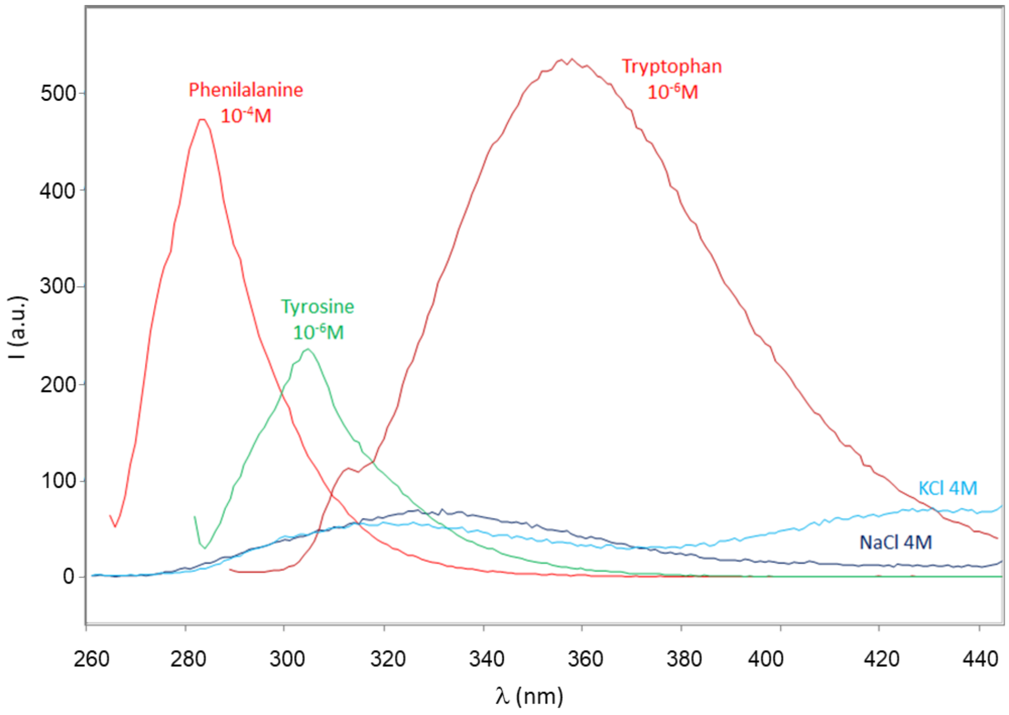

Figure S4. Fluorescence emission spectra of 4M KCl and NaCl aqueous solutions (excitation at 227 nm), phenylalanine 10^{-4}M (excitation at 257 nm), tyrosine 10^{-6}M (excitation at 275 nm), and tryptophan 10^{-6}M (excitation at 280 nm). Spectra were collected under the same instrumental conditions.

3. KCl water solutions fluorescence vs concentration.

The fluorescence intensity of the 310 nm emission band was measured at concentrations from 1.28 mM to 4M in water. As can be seen in Fig.S5, above 1M the emission intensity increase seems to reach a plateau.

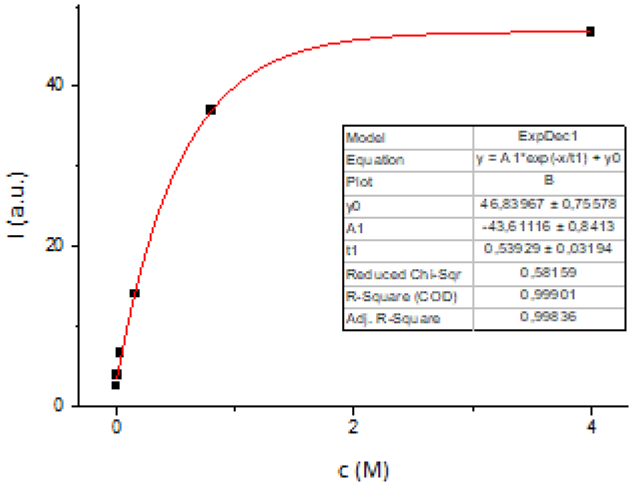

FIGURE S5. Fluorescence intensity of the 310 nm band (exc = 227 nm) as a function of KCl concentration in water.

4. Fluorescence emission spectra of NaCl water solutions vs concentration.

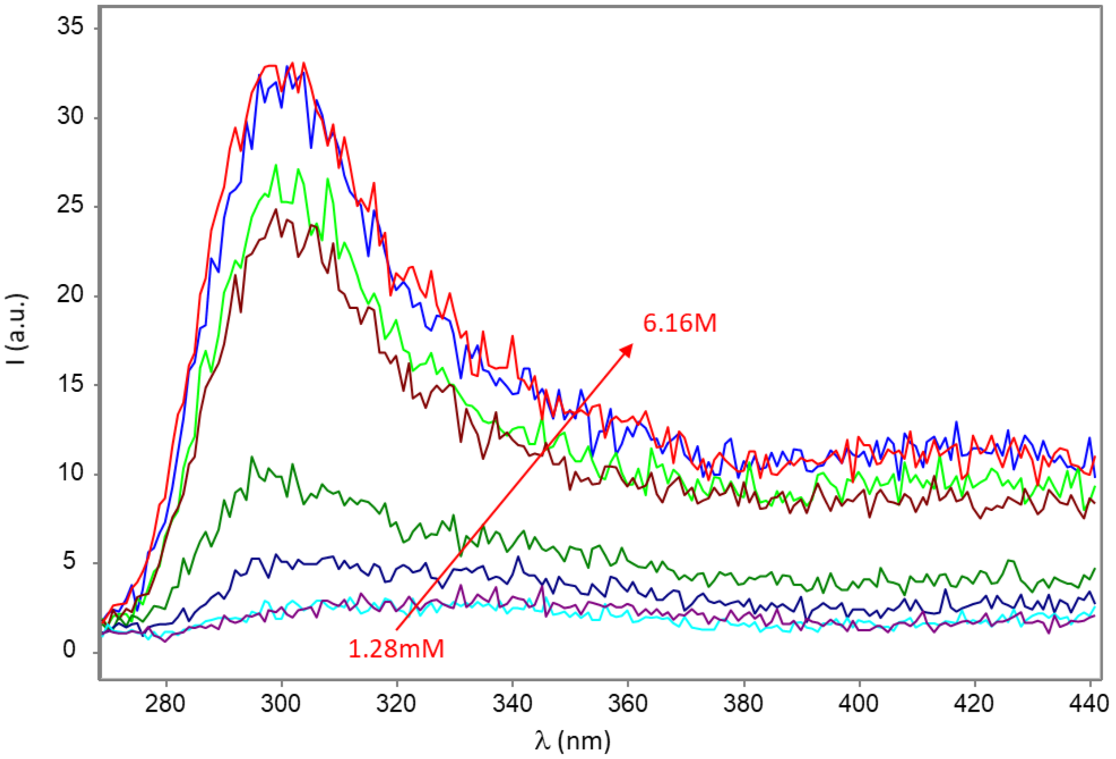

FIGURE S6. Fluorescence emission spectra of NaCl aqueous solutions at different concentrations from 1.28 mM to 6.16M (saturated solution).

5. Fluorescence emission intensity of 4M KCl solutions vs temperature

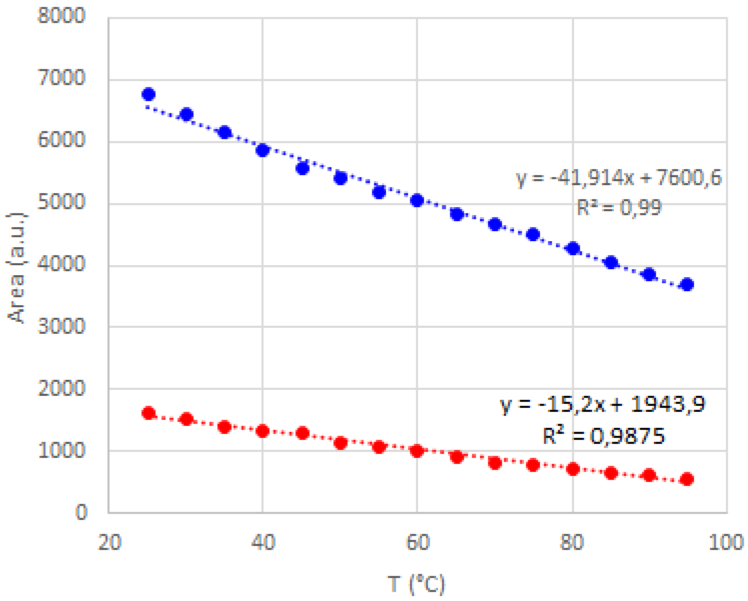

FIGURE S7. Fluorescence emission intensity of 4M KCl water solutions at different temperatures from 25°C to 95°C. Red: 310 nm band area with excitation at 227 nm; blue: 430 band area with excitation at 240 nm.

6. Activation energy for radiationless decay.

The fluorescence quantum yield $\Phi_f(T)$ is given by:

$$\Phi_f(T) = \frac{k_f}{k_f + k_d} \tag{1}$$

where k_d is the sum of the rate constants corresponding to the processes that quench fluorescence and lead to radiationless deactivation. k_d is function of temperature with the form:

$$k_d(T) = k_d^0 exp\left(-\frac{\Delta E_a}{RT}\right) \tag{2}$$

Combining these equations we obtain:

$$ln\left(\frac{1}{\Phi_f(T)} - 1\right) = -\frac{\Delta E_a}{RT} + ln\left(\frac{k_d^0}{k_f}\right) \tag{3}$$

where $\Phi_f(T)$ is the quantum yield and ΔE_a is the activation energy for the temperature dependent radiationless deactivation of the excited fluorescent states.

In the limit of low values of $\Phi_f(T)$, we can neglect the term -1 in the argument of the logarithm in eq. (3) and obtain the simplified equation [5]:

$$ln\left(\frac{1}{I_N(T)}\right) = \frac{-\Delta E_a}{RT} + lnB \quad (4)$$

where I(T) is the measured fluorescence intensity at temperature T. We assume that the unknown proportionality constant between Φ_f(T) and I_f(T) does not depend on T, so B is a constant. Indeed this assumption is safe since we observe linear Arrhenius kinetics. From eq. (4), the slope of the straight line obtained by plotting $ln\left(\frac{1}{I_N(T)}\right)$ versus 1/T (where T is expressed in kelvin) gives the value of the activation energy.

7. Fluorescence emission spectra of 4M NaCl water solutions vs temperature.

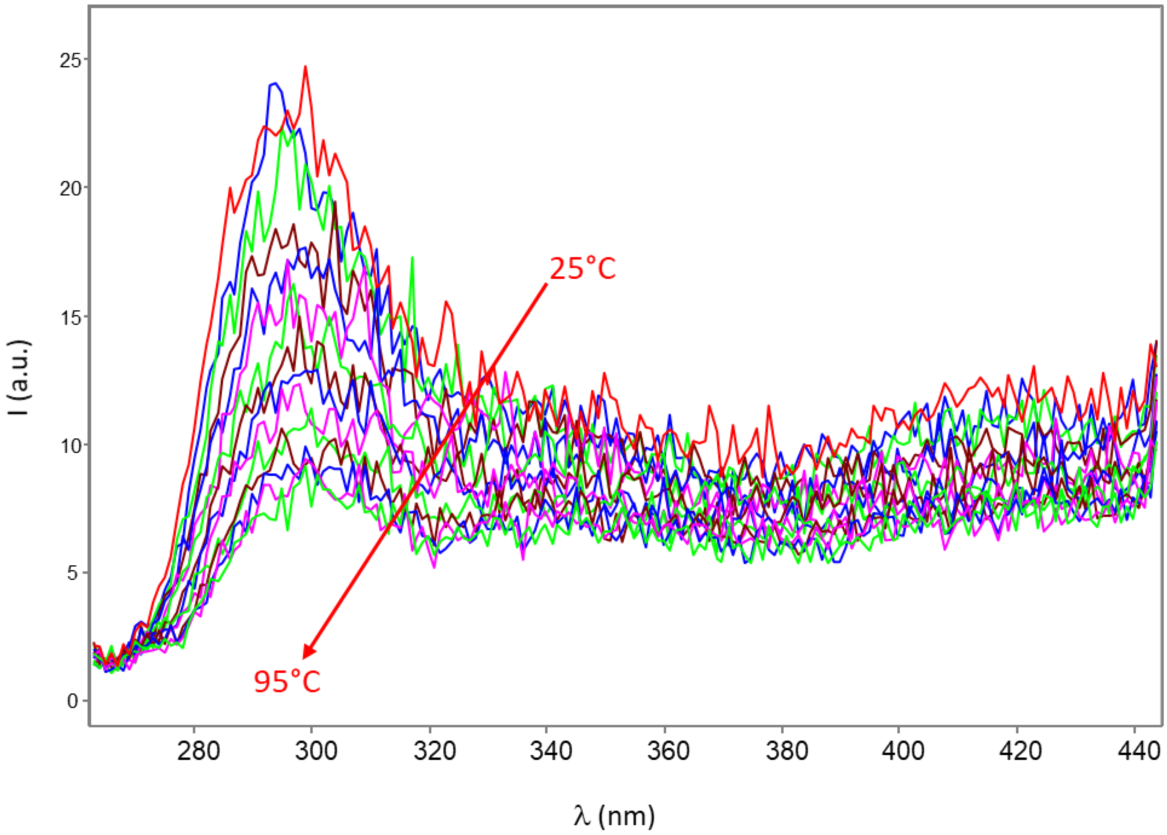

Figure S8. Fluorescence emission spectra of 4 M NaCl aqueous solutions at different temperatures, from 25°C to 95°C; excitation at 227 nm. Conditions: spectrofluorometer, Varian Cary Eclipse; photomultiplier gain, 900 V; excitation slit, 5 nm; emission slit, 5 nm.

8. Fluorescence emission spectra of milliQ H_2O and KCl 4M

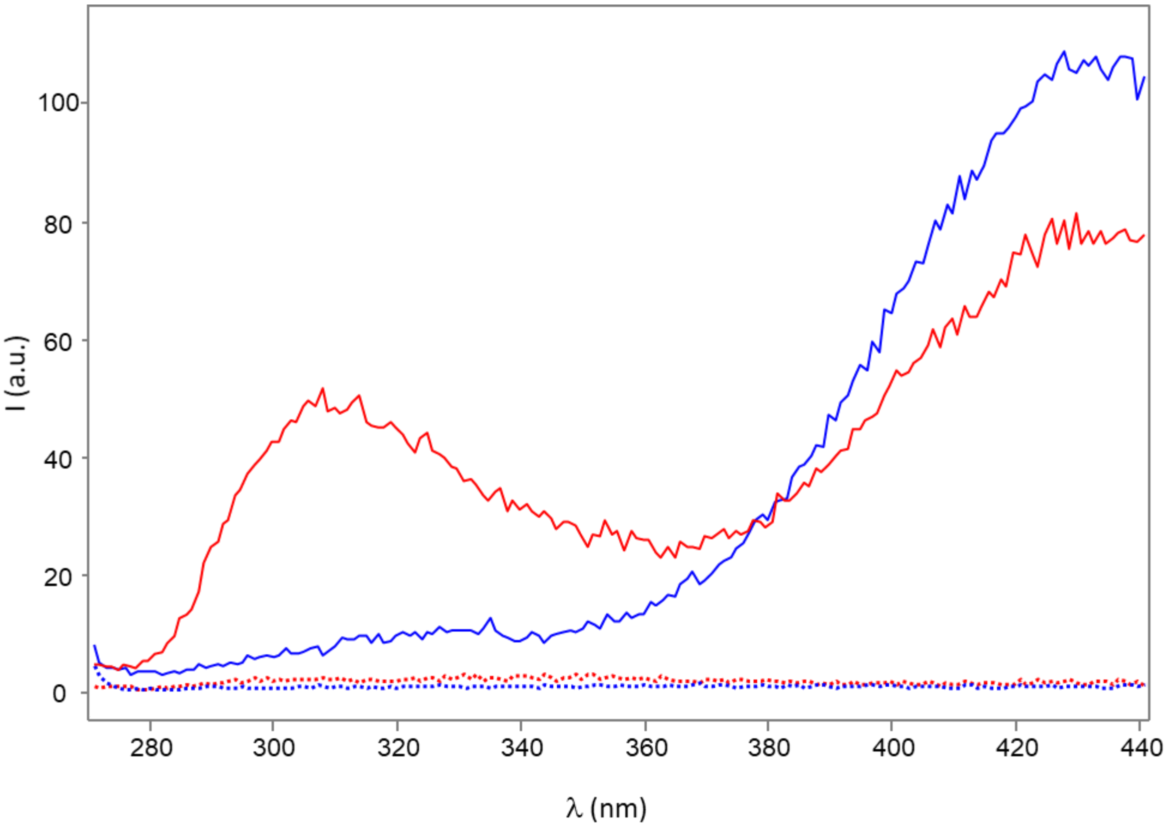

Figure S9. Fluorescence emission spectra of 4M KCl aqueous solutions (continuous line) and of H_2O milliQ (dotted lines). Red: excitation at 227 nm. Blue: excitation at 240 nm. Conditions: spectrofluorometer, Varian Cary Eclipse; photomultiplier gain, 900 V; excitation slit, 5 nm; emission slit, 5 nm.

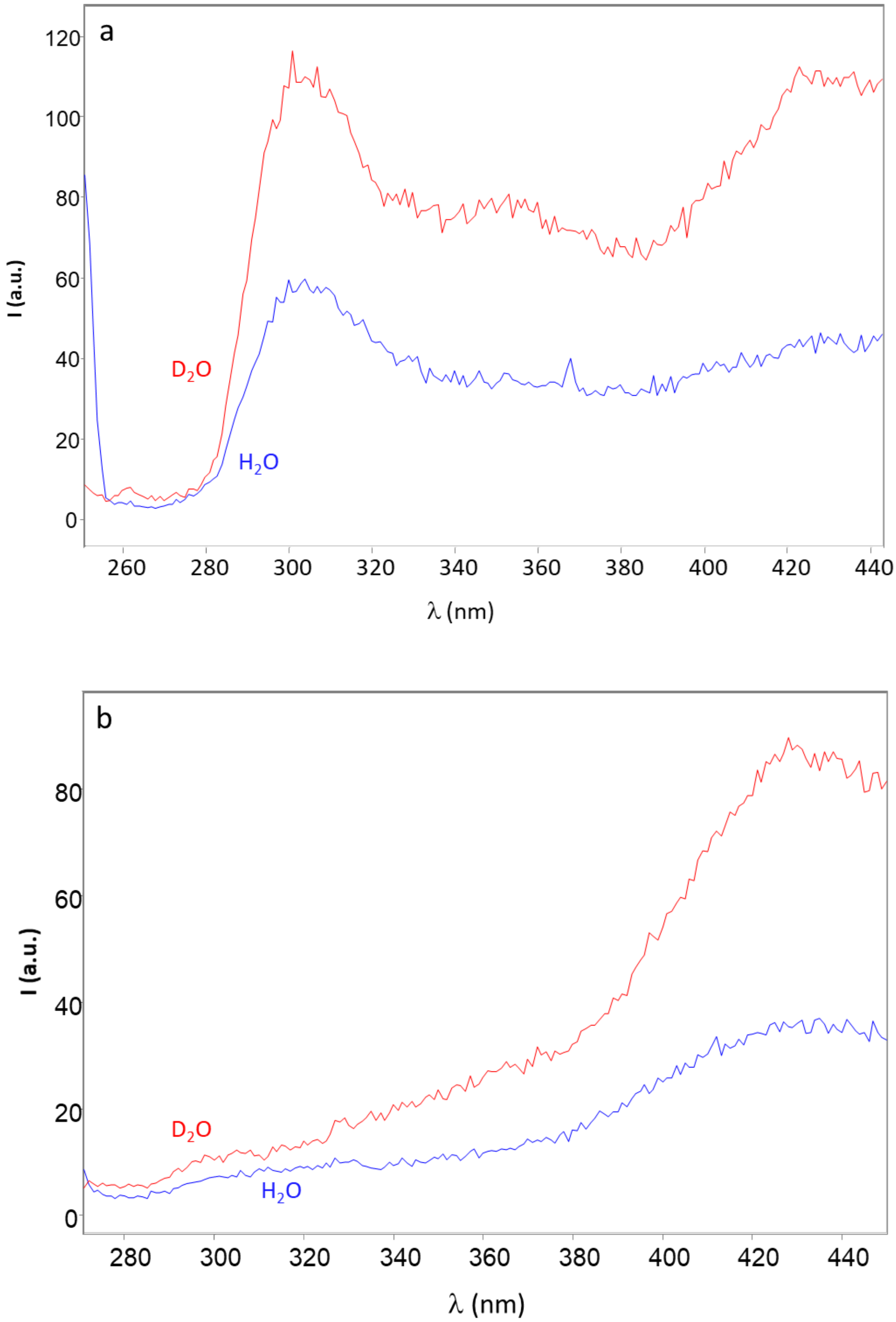

Figure S10. Fluorescence emission spectra of 2 M KCl aqueous solutions in H_2O (blue) and in D_2O (red); a) excitation at 227 nm; b) excitation at 240 nm. Conditions: spectrofluorometer, Varian Cary Eclipse; photomultiplier gain, 900 V; excitation slit, 5 nm; emission slit, 5 nm.

9. Computations.

Classical mechanism protocol. Merck molecular force field 94x (MMFF94x) has been selected for molecular dynamic (MD) simulation since it is well suited to describe the counterions. A 100 ns MD run at 298K in gas-phase has been carried out with 0.5 fs time step. In order to select ion buried forms, a centroid spherical wall restrain is set within a restrained MD approach. We saved a structure every 20 ps. These structures are successively energy minimized and analyzed as a function of the ion positions. We select the starting points for DFT geometry optimizations with different ion-ion geoetries among those 1) with ions buried in the solvent; 2) lower in energy.

DFT and TDDFT. All the computations presented here have been carried out using TURBOMOLE suite of programs, using pure Gradient Generalized Approximation B-LYP functional. The Resolution of Identity (RI) technique was adopted in order to save CPU time. Basis sets of triple-ζ plus polarization split valence quality (def-TZVP) were adopted for all the atoms included in the systems under investigation. DFT grid-size was set to standard m3 value. Ground state geometry optimizations were carried out with convergence criteria fixed to 10^{-6} hartree for the energy and 0.001 hartree·bohr^{-1} for the gradient norm vector. Electronic spectra have been obtained first generated by TDDFT computations and and then convoluted (solid line) using oscillator strength weighted Gaussian distribution functions centered on the computed excitation energies (nm) with half widths at half-maxima of 70 nm.

In general the choice of the cluster structure for TD-DFT computations is the result of an exploration of the cluster potential energy surface as follows. In a 100 ns molecular dynamic we identified a number of geometries which are representative of the possible configuration of the cluster. In particular, six different configurations can be identified according to the scheme below:

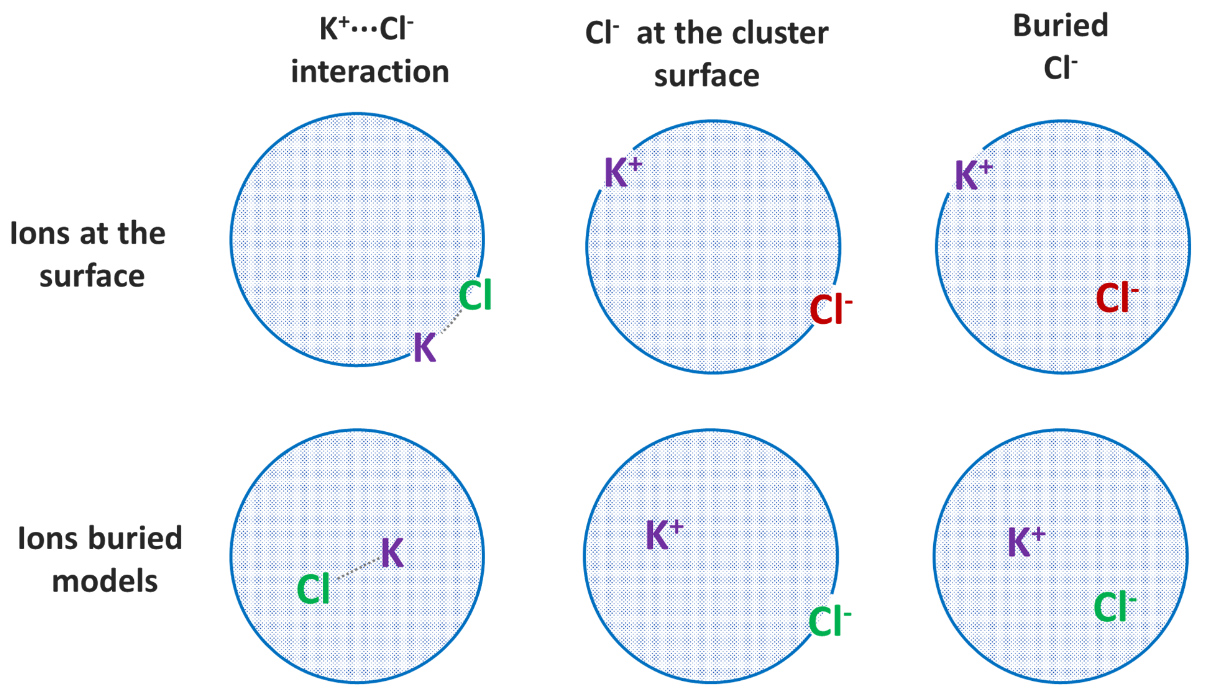

When two KCl are included in the model cluster, we can generally distinguish among structures in which

1) ions are free or, at least, forming solvent separated ion-pairs;
2) we observe the formation of ion-ion interactions only between one K+ and one Cl- at a time
3) We observe the formation of a "dimer" ion water cluster, in which the four ions interact with each other.

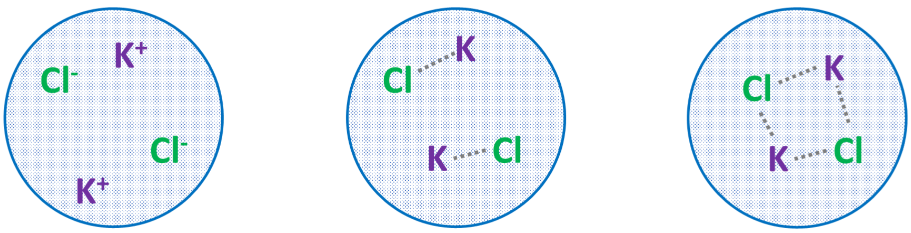

DFT optimization protocol. Starting from the set of structures selected with restrained MD simulation, we fully optimize their geometry at DFT level. At the end of DFT optimizations, we analyzed and classified the structures as i) ions buried in the solvent; ii) ions exposed at the surface clusters. These latter have not been considered for further TD-DFT investigations.

Table S1. $KCl(H_2O)_{54}$ buried ions models. ΔE is the b-lyp/TZVP energy difference in kcal/mol; $d(K^+{\cdot}Cl^-)$ is the ion-ion distances Å; CN K^+ and CN Cl^- are the coordination number around the ions (distance threshold equal to 4Å); $<K^+>$ and $<Cl^->$ are the average K-O and Cl-H interatomic distances (in Å) between the ion and the nearest neighbors atoms that belong to the ion coordination sphere; NH_b and $<H_b>$ are the total number of H-bond interaction and the corresponding average O-H distance (in Å); 1ex is the band assignment according to the population of the HOMO->LUMO monoelectronic transition and $E(S_1)$ is the excitation energy in nm at the ground state geometry. In these models, the K^+ ion is on pentacoordinated with an average K-O distance of 3.09±0.21Å, while Cl^- is six-coordinated with average Cl-H distance of 2.31±0.21Å (experimental values 2.90 and 2.35Å respectively).[6] The average is number of H_2O molecules around ions are 7.3 (K^+) and 6.0 (Cl^-).

	**ΔE**	**$d(K^+\text{-}Cl^-)$**	**CN K^+**	**CN Cl^-**	**$<K^+>$**	**$<Cl^->$**	**NH_b**	**$<H_b>$**	**1ex**	**$E(S_1)$**
1	0,0	4,125	9	6	3,222	2,308	85	1,802	O→K,H	268,3
2	4,1	4,237	7	6	3,118	2,294	85	1,803	O→K,H	243,7
3	9,2	3,896	7	6	3,063	2,320	90	1,850	O→K,H	254,7
4	10,1	3,672	7	6	3,083	2,283	90	1,848	O→K,H	256,9
5	11,0	7,079	7	6	3,227	2,258	87	1,836	O→K,H	244,8
6	11,1	6,560	8	7	3,105	2,329	87	1,839	O→K,H	264,1
7	14,0	6,649	8	7	3,120	2,371	83	1,805	O→K,H	252,4
8	17,2	4,575	8	7	3,058	2,310	89	1,833	O→K	246,9
9	20,2	3,963	6	6	3,092	2,301	84	1,810	O→K	258,3
10	20,9	4,433	9	5	3,234	2,190	91	1,869	O,Cl→K	252,6
11	22,4	5,909	12	5	3,348	2,222	88	1,843	O→K,H	243,4
12	22,9	3,184	5	6	3,128	2,295	89	1,846	O→K,H	249,7
13	23.7	3,444	4	6	3.086	2.312	84	1.808	O→K,H	276,1
14	26,2	3,196	4	7	3,217	2,222	85	1,820	O→K,H	264,0
15	33.9	4.847	6	8	3.109	2.297	85	1,837	O→K,H	287,6
16	42,8	6.008	6	8	3,161	2,386	82	1,833	O→K,H	284,5
17	43.4	3,399	4	9	3,314	2,287	89	1,865	O→K	243,5
18	45.8	4,845	6	8	3,121	2.258	88	1.874	O→K,H	286,3

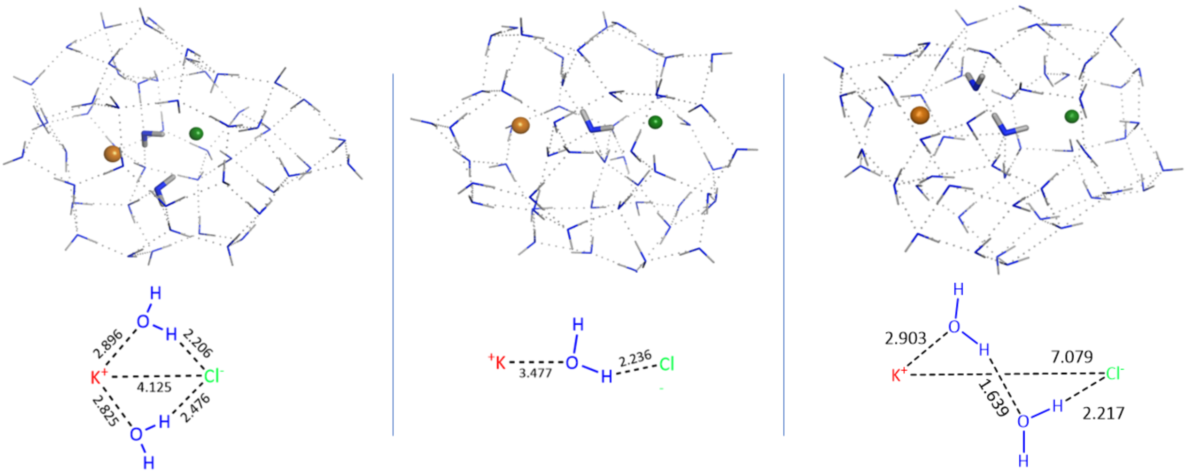

Figure S11. 1M KCl models. For each structure the corresponding ion configuration is sketched (distances in Å). Going from left to right are reported low energy KCl/water structures that model solvent shared and solvent separated ion-pairs (model 1, 5 and 6 from Table S1 in SI).

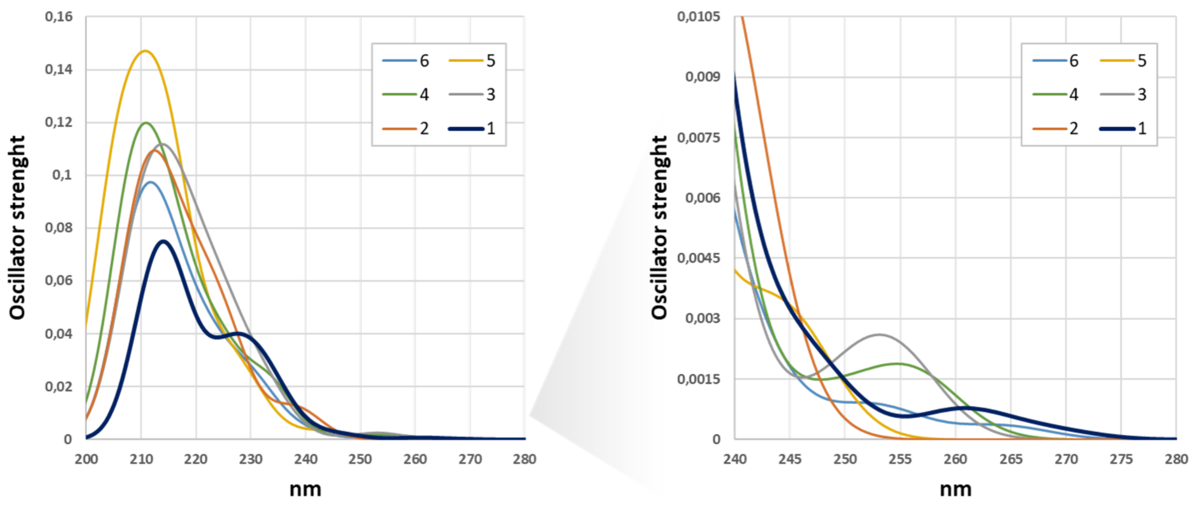

Figure S12. Computed absorption spectra (on the left) for the first six most stable KCl 1 M models ($KCl(H_2O)_{54}$). Stick spectra were first generated and then convoluted (solid line) using oscillator strength weighted Gaussian distribution functions centered on the computed excitation energies (nm) with halfwidths at half-maxima of 40 nm. On the right the is put in evidence the computed lowest energy spectral region.

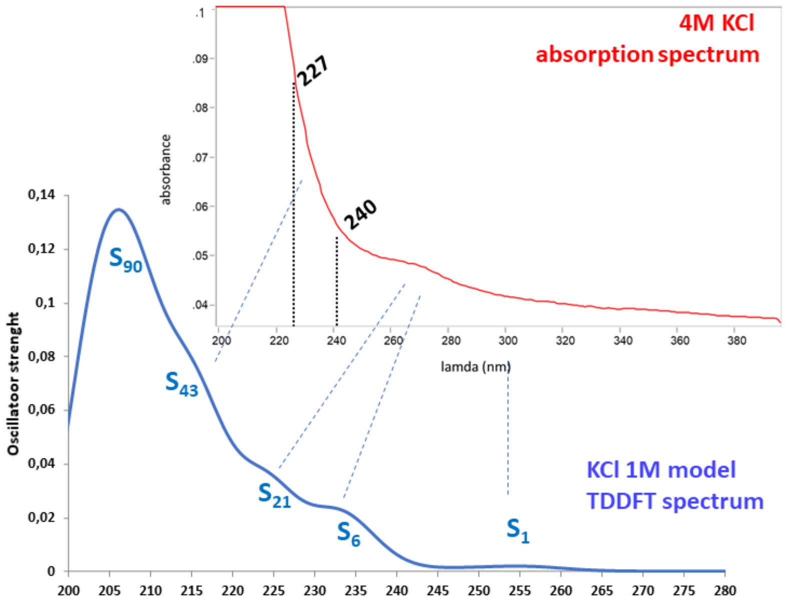

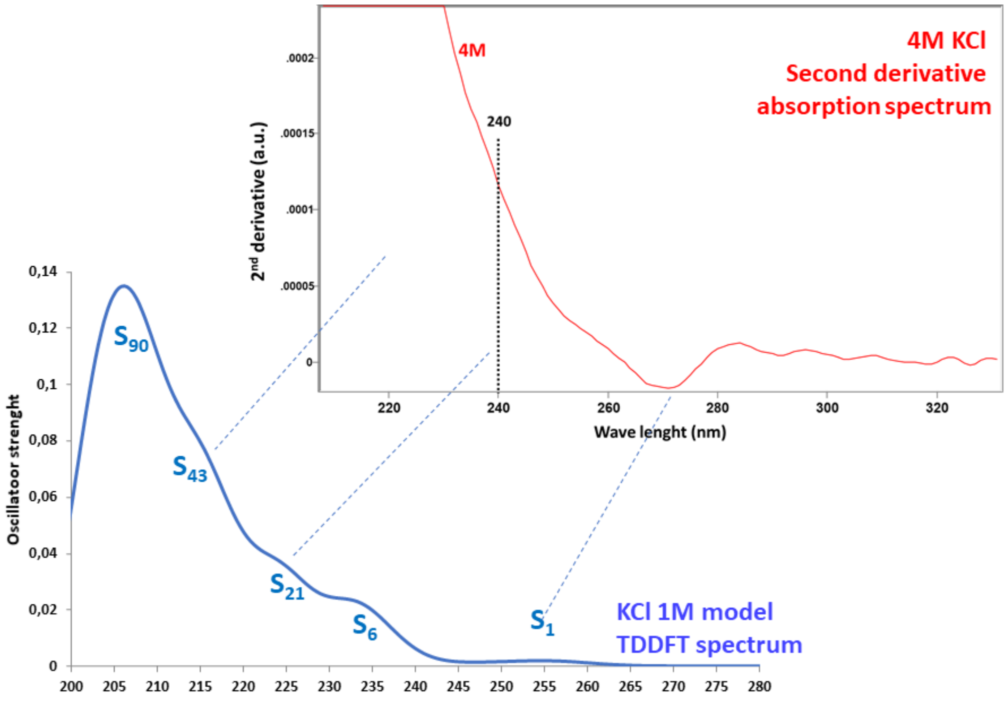

ex	1e	%	f	CT
1	HOMO→LUMO	100	3·10-4	O→K
6	HOMO-1→LUMO+1	100	1·10-4	O→K
21	HOMO-3→LUMO+1	100	2·10-4	O→K
43	HOMO-1→LUMO+4	100	0.0013	O→H
90	HOMO-1→LUMO+6<br>HOMO-10→LUMO+2	79<br>19	0.012	O→H

Figure S13. TDDFT Computed absorption spectrum for KCl 1 M models ($KCl(H_2O)_{54}$ model 1 from Table S1) compared with experimental 4M KCl absorption and second derivative absorption spectra. In the table below the figure are reported the relevant 1e mono-electronic transitions and oscillator strength computed at TDDFT level. By comparing the spectra, the weak shoulder measured at 260-270 nm is tentatively assigned to the computed weak shoulder around 230-235 nm, which corresponds to S_6 excitation.

Table S2. TDDFT/TZVP Excitation energy in nm for S_1 state at the ground state geometry $KCl(H_2O)_{54}$ buried ion isomers 1 and 6 (see Table S1) as a function of the DFT functional adopted. In B-LYP+disp3 dispersion corrections were also included using the D3 approach. In all the cases S1 mono-electronic transition is HOMO->LUMO with a O->K,H CT character.

	**1**			**5**		
	**d(K^+-Cl^-)**	**<Hb>**	**E(S_1)**	**d(K^+-Cl^-)**	**<Hb>**	**E(S_1)**
**B-LYP**	4.125	1.801±0.105	268.3	7.079	1.847±0.136	244.8
**B-LYP + disp3**[7]	3.782	1.766±0.097	267.1	6.698	1.808±0.114	243.6
**B3LYP**[8,910]	4.092	1.806±0.100	193.8	7.028	1.854±0.134	184.6
**cam-B3LYP**[18]	4.106	1.765±0.097	163.0	6.939	1.812±0.121	158.1
**BP86**[8,11]	4.089	1.735±0.105	247.9	6.975	1.781±0.145	233.3
**PBE**[12]	4.032	1.738±0.110	261.3	6.954	1.793±0.141	240.5
**PBE0**[13]	4.045	1.767±0.106	178.6	6.963	1.817±0.136	172.1
**TPSS**[14,15,16]	3.949	1.760±0.107	242.4	6.968	1.798±0.123	223.2
**TPSSh**[17]	4.017	1.775±0.103	207.1	6.991	1.816±0.120	194.9

Table S3. Differential LUMO-HOMO atomic mulliken populations for the atoms involved in charge transfer in S_1 excitation for $KCl(H_2O)_{54}$ model 1-3, 5 and 6, as reported in Table S1. For a given atomic species A, The δ values are a semi-quantitative esteem of the CT upon S1 excitation and are computed as Q(A,LUMO)-Q(A,HOMO). The negative sign and positive sign indicate a shift or a gain in electron population respectively.

	**ΔE**	**d(K^+-Cl^-)**	**E(S_1)**	**δO**	**δCl**	**δK**	**δH**
1	0,0	4,125	268,3	0,51	-0.01	-0,47	-0,12
2	4,1	4,237	243,7	0,54	+0.01	-0,54	-0,06
3	9,2	3,896	254,7	0,39	-0.01	-0,16	-0,11
5	10,1	3,672	256,9	0,44	-0.01	-0,14	-0,13
6	11,0	7,079	244,8	0,51	+0.01	-0,37	-0,07

Table S4. TDDFT S_1 geometry optimizations for $KCl(H_2O)_{54}$ structures from Table S1. $E(S_1)$ is the vertical excitation energy; E(opt kcl) is the final excitation energy when only K and Cl atoms are left free to move; All excitation energies in nm. Atomic charges q are reported for K and Cl for the S_0 ground state and S_1 in electrons. Δq are the S_1-S_0 atomic charge differences. In the two pictures below this table are sketched the structures of a cluster in which are evidenced as stick-and-ball the active atoms during TDDFT optimization.

n	1ex	E(S1)	opt S1 kcl	Δλ
1	O→K,H	268,3	275,7	7,4
2	O→K,H	243,7	250,5	6,8
3	O→K,H	254,7	298,1	43,4
4	O→K,H	256,9	293,9	37
5	O→K,H	244,8	256,3	11,5
6	O→K,H	264,1	284,9	20,8
7	O→K,H	252,4	278,4	26
8	O→K	246,9	251,5	4,6
9	O→K	258,3	261,9	3,6
10	O,Cl→K	252,6	1016	Cl
11	O→K,H	243,4	275,8	32,4
12	O→K,H	249,7	278,5	28,8
13	O→K,H	276,1	290,4	14,3
14	O→K,H	264,0	290,8	26,8
15	O→K,H	287,6	296,5	8,9
16	O→K,H	284,5	305,9	21,4
17	O→K	243,5	254,2	10,7
18	O→K,H	286,3	302,2	15,9

n	$q(K,S_0)$	$q(K, S_1)$	Δq(K)	$q(Cl\ S_0)$	$q(Cl, S_1)$	Δq(Cl)
1	0,78	0,30	0,48	-0,66	-0,65	0,01
2	0,79	0,25	0,54	-0,67	-0,66	0,01
3	0,76	0,60	0,17	-0,65	-0,66	-0,01
4	0,76	0,62	0,14	-0,65	-0,66	-0,01
5	0,76	0,39	0,37	-0,67	-0,66	0,01
6	0,77	0,65	0,12	-0,68	-0,66	0,02
7	0,78	0,58	0,20	-0,67	-0,65	0,02
8	0,75	0,15	0,59	-0,66	-0,63	0,03
9	0,78	0,20	0,57	-0,67	-0,65	0,02
10	0,76	0,32	0,44	-0,63	-0,63	0,00
11	0,78	0,23	0,56	-0,65	-0,65	0,01
12	0,80	0,13	0,67	-0,69	-0,68	0,02
13	0,77	0,49	0,28	-0,69	-0,68	0,02
14	0,77	0,42	0,34	-0,69	-0,67	0,02
15	0,79	0,27	0,51	-0,66	-0,66	0,00
16	0,78	0,69	0,09	-0,67	-0,65	0,02
17	0,75	0,13	0,62	-0,68	-0,68	0,00
18	0,75	0,73	0,02	-0,65	-0,64	0,01

Table S5. $(KCl)_2(H_2O)_{53}$ ions models. ΔE is the B-LYP/TZVP energy difference in kcal/mol; <K^+•Cl^-> is the average ion-ion distance Å; S_1 is the band assignment according to the population of the HOMO->LUMO monoelectronic transition (1ex) and $E(S_1)$ is the excitation energy in nm at the ground state geometry. $(KCl)_2(H_2O)_{53}$ models the average K-Cl distance is 4.06±0.58Å.

	**d(K^+-Cl^-)**	**ΔE**	**1ex**	**nm**
1	3.388	0.0	Cl→K	274,8
2	3.841	4.0	O→K	261,8
3	4.023	6,2	O→K	273,6
4	4.189	8,8	Cl→K, H	284,9
9	3.401	11,7	O→K	253,2
5	3.990	14,5	Cl→K	262,3
6	3.710	14,7	O→K	269,7
7	3.779	14,8	Cl→K	256,5
8	4.710	16,8	Cl→K, H	289,7
10	4.703	18.0	Cl→K	290,7
11	3.885	18,4	O Cl→K	242,5
12	3.863	18,5	O→K	261,5
13	3.616	19,8	O→K,H	308,6
14	3.459	21,7	O→K	285,4
15	3.946	22,5	Cl→K	306,3
16	4.507	23,9	O→K,H	279,6
17	3.414	24,7	O→K	264,9
18	4.179	24,8	O→K	263,4
19	4.699	29,6	Cl→K	279,5
20	3.678	30,7	O→K,H	294,4
21	3.711	31,8	O→K	281.0
22	5.798	32,3	Cl→K, H	279,1
23	4.781	33,5	O→K	253,3

Tables S6. $(KCl)_2(H_2O)_{108}$ ions models. ΔE is the B-LYP/TZVP energy difference in kcal/mol; in the column type is reported the type of cluster obtained according to the ion positions among each other (2K+2Cl- ion not directly interacting; KCl+KCl ions interact as two salt couple; (KCl)2 ions forms square or tetrahedral cores; K2Cl+Cl- ions form a triad K2Cl+ and the second Cl- results not interacting with the triad, see scheme at the bottom of this table) $d(K^+{\cdot}Cl^-)$, $d(K^+{\cdot}K^+)$ and $d(Cl^-{\cdot}Cl^-)$ are the various ion-ion distances Å;Σ CN K and Σ CN Cl are the number of K-O and Cl-H interaction respectively with threshold equal to 3Å; NH_b and <H_b> are the total number of H-bond interaction and the corresponding average O-H distance (in Å); S_1 is the band assignment according to the population of the HOMO->LUMO monoelectronic transition and $E(S_1)$ is the excitation energy in nm at the ground state geometry. S_1 opt kcl is the excitation energy (in nm) at the local minimum on the S_1 PEs upon TDDFT constrained geometry optimization; Δλ is the difference between emission and S_1 absorption excitation energy wavelengths (in nm). CI is for conical intersection, when during TD-DFT optimization, the geometry reaches the point R in which $E(S_1)=E(S_0)$. K-Cl average distance is 5.86±1.37Å, while K-K and Cl-Cl values are 6.50±1.98Å and 10.08±2.24Å. The average coordination number of the ions are 7.2 (K^+) and 5.4 (Cl^-).

	$(KCl)_2(H_2O)_{108}$108	ΔE	K-Cl				K-K	Cl-Cl	CN K+	CN Cl-	nH_b	<H_b>
1	2K+2Cl-	0,0	5,6	5,4	8,1	8,5	6,7	8,1	11	11	174	1,803
2	2K+2Cl-	6,3	3,4	3,6	5,5	5,5	6,2	6,8	9	12	183	1,836
3	2K+2Cl-	6,8	3,4	3,6	5,5	5,5	6,2	6,8	9	12	183	1,837
4	(KCl)2	11,1	3,4	3,5	3,6	5,4	6,1	4,9	9	13	183	1,831
5	KCl + KCl	12,9	3,3	3,5	5,6	5,6	6,2	7,0	10	13	184	1,845
6	2K+2Cl-	15,6	5,1	5,7	7,7	11,0	10,5	13,5	10	10	174	1,808
7	(KCl)2	16,9	3,2	3,3	3,4	4,4	4,5	5,2	7	11	189	1,844
8	K+(KCl)Cl-	17,1	3,2	4,8	5,7	8,0	5,0	5,8	9	11	178	1,825
9	K+(KCl2)-	17,1	3,2	4,9	5,7	8,0	5,0	5,9	9	11	178	1,825
10	K2Cl+Cl-	17,8	3,3	3,4	4,5	6,1	4,5	6,8	7	13	177	1,812
11	(KCl)2	19,6	3,2	3,3	3,9	4,6	4,5	5,5	7	7	187	1,841
12	K2Cl+Cl-	20,0	3,3	3,3	4,4	6,1	4,5	6,6	7	10	177	1,813
13	(KCl)2	23,9	3,3	3,3	3,6	4,4	4,7	5,2	7	10	188	1,844
14	(KCl)2	24,0	4,6	4,8	4,8	5,5	4,9	7,4	10	12	177	1,819
15	K2Cl+Cl-	26,8	3,3	3,3	5,1	5,3	4,7	6,8	8	11	171	1,802
16	(KCl)2	30,7	3,3	3,3	3,3	4,0	4,8	4,7	8	10	192	1,877
17	(KCl)2	31,1	6,1	6,2	6,3	10,3	8,8	10,1	7	11	178	1,817
18	2K+2Cl-	34,6	6,1	6,3	7,5	10,4	5,7	5,5	10	12	179	1,851
19	2K+2Cl-	35,7	6,6	6,9	7,2	7,4	7,9	8,0	10	12	177	1,832
20	2K+2Cl-	35,8	4,9	5,4	5,4	7,3	6,8	8,8	10	12	176	1,826
21	(KCl)2	35,8	3,2	3,4	3,5	4,0	4,9	9,1	7	10	180	1,821
22	(KCl)2	36,2	3,2	4,3	4,5	4,9	4,9	6,7	7	11	174	1,812
23	KCl + KCl	36,9	3,4	5,9	10,0	12,1	10,4	13,2	9	10	175	1,819
24	2K+2Cl-	37,7	5,4	5,7	6,1	6,3	6,4	97,0	11	12	178	1,835
25	2K+2Cl-	42,1	5,7	6,2	6,6	6,7	6,6	10,7	9	12	175	1,823
26	(KCl)2	45,4	3,3	3,4	4,5	5,2	5,4	5,2	8	9	173	1,804
27	2K+2Cl-	56,6	4,7	4,9	6,8	7,1	5,8	5,9	10	10	175	1,833

28	2K+2Cl-	56,9	3,5	4,7	5,2	5,7	5,1	7,2	10	12	178	1,847
29	2K+2Cl-	62,3	5,4	5,9	7,0	8,1	10,7	7,5	12	11	179	1,851
30	2K+2Cl-	77,2	4,8	6,0	6,8	8,3	9,5	8,5	11	11	172	1,828

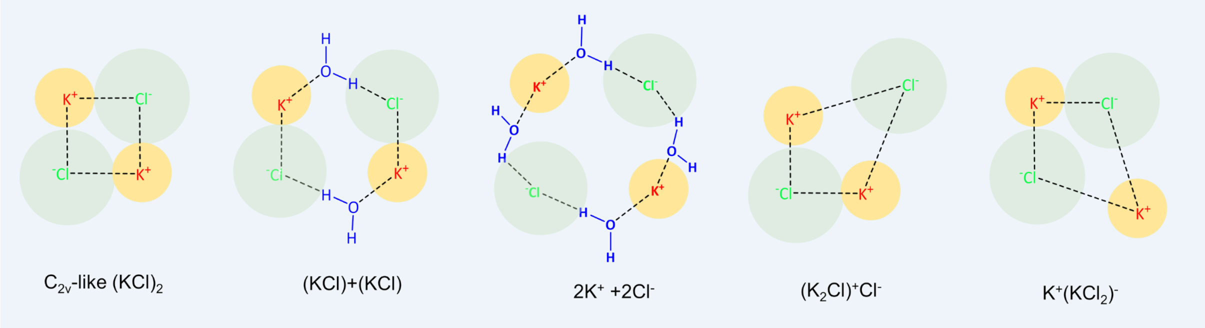

	$(KCl)_2(H_2O)_{108}$	ΔE	1ex	E(S1)	S1 opt kcl	Δλ
1	2K+2Cl-	0,0	O→H	312,0	321,1	9,1
2	2K+2Cl-	6,3	O→K	279,8	288,4	8,6
3	2K+2Cl-	6,8	O→K	279,8	288,6	8,8
4	(KCl)2	11,1	O→H	300,4	328,0	27,6
5	KCl + KCl	12,9	O→K,H	277,0	283,9	6,9
6	2K+2Cl-	15,6	O→H,K	329,9	344,8	14,9
7	(KCl)2	16,9	Cl→K	265,3	397,4	132,1
8	k+(KCl)Cl-	17,1	O→K	271,5	288,2	16,7
9	k+(KCl)Cl-	17,1	O→K	271,4	288,2	16,8
10	K2Cl+Cl-	17,8	O→K,H	259,3	CI	
11	(KCl)2	19,6	Cl→K	272,3	380,5	108,2
12	K2Cl+Cl-	20,0	O,Cl→K,H	258,7	CI	
13	(KCl)2	23,9	Cl→H,K	267,9	376,3	108,4
14	(KCl)2	24,0	O→K,H	315,6	389,1	73,5
15	K2Cl+Cl-	26,8	O→K	269,4	303,2	33,8
16	(KCl)2	30,7	O→K	257,3	271,2	13,9
17	(KCl)2	31,1	O→K	290,8	304,9	14,1
18	2K+2Cl-	34,6	O→K,H	285,4	329,4	44,0
19	2K+2Cl-	35,7	O→K,H	273,7	280,6	6,9
20	2K+2Cl-	35,8	O→H,K	276,1	297,3	21,2
21	(KCl)2	35,8	Cl→K	262,3	351,6	89,3
22	(KCl)2	36,2	O→K,H	301,0	308,3	7,3
23	KCl + KCl	36,9	O→K,H	276,0	428,5	152,5
24	2K+2Cl-	37,7	O→H,K	295,3	307,9	12,6
25	2K+2Cl-	42,1	O→K,H	281,6	297,4	15,8
26	(KCl)2	45,4	O→K	263,9	CI	
27	2K+2Cl-	56,6	O→K	301,9	314,1	12,2
28	2K+2Cl-	56,9	O→K	287,8	291,7	3,9
29	2K+2Cl-	62,3	O→H	312	325,3	13,3
30	2K+2Cl-	77,2	O→K,H	290,4	323,3	32,9

Table S7. Differential LUMO-HOMO atomic mulliken populations for the atoms involved in charge transfer in S_1 excitation for model 1,4,7 and 8, as reported in Table S6. The negative sign and positive sign indicate a shift or a gain in electron population respectively.

**n**	type	S1 CT		
1	2K+2Cl-	O→H	4O (-0.8 e)	5H (+0.5 e)
4	2K+2Cl-	O→K	3O 1H (-0.7 e)	2K (+0.6 e)
7	(KCl)2	Cl→K	Cl(-0.6 e)	2K (+0.5 e)
8	(KCl)2	Cl→K	Cl(-0.6 e)	2K (+0.5 e)

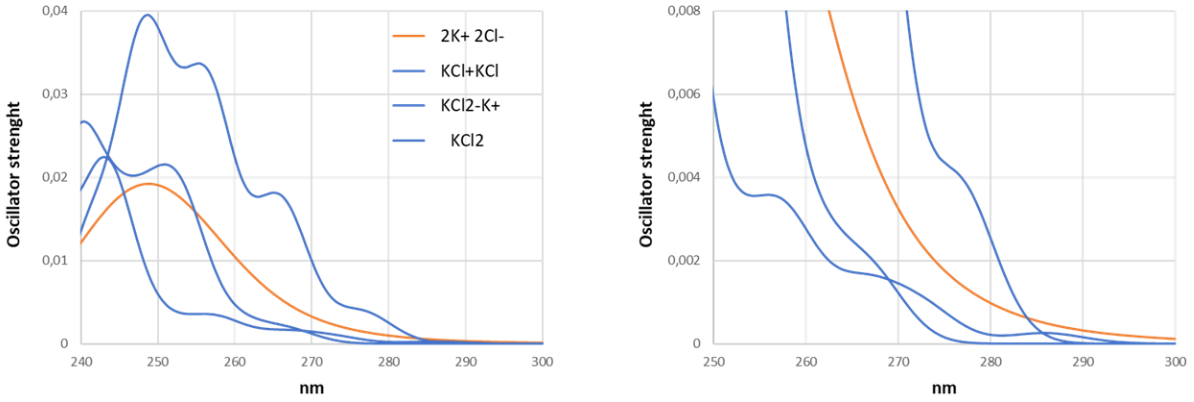

Figure S13. Computed absorption spectra (on the left) for the most stable KCl 1 M models $(KCl)_2(H_2O)_{108}$ according to the ion reciprocal position. Stick spectra were first generated and then convoluted (solid line) using oscillator strength weighted Gaussian distribution functions centered on the computed excitation energies (nm) with halfwidths at half-maxima of 40 nm. On the right the is put in evidence the computed lowest energy spectral region. According to this series of KCl/water cluster models, experimental S1 excitation energy is estimated at 305 nm.

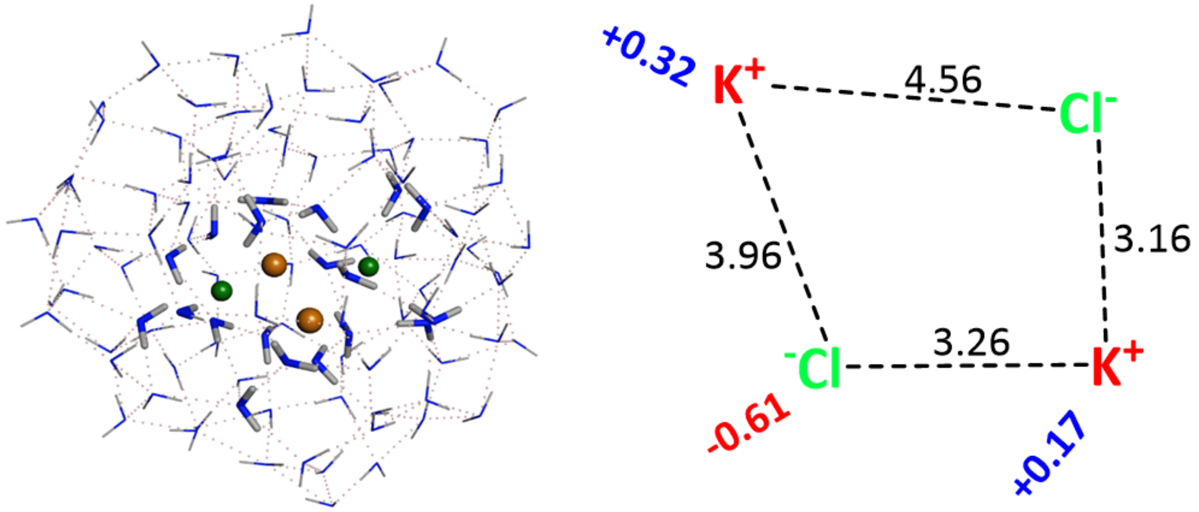

Figure S14. Optimized $(KCl)_2(H_2O)_{108}$ model 7 from Table S6. On the left the structure is reported, evidencing the ion positions and the water molecules around them. On the right are reported the ion-ion distances in (in Å) and the computed S_1 charge transfer according to the Mulliken atomic population differences between S_1 and S_0 atomic charges computed at the charge S_0 minimum geometry (in electrons).

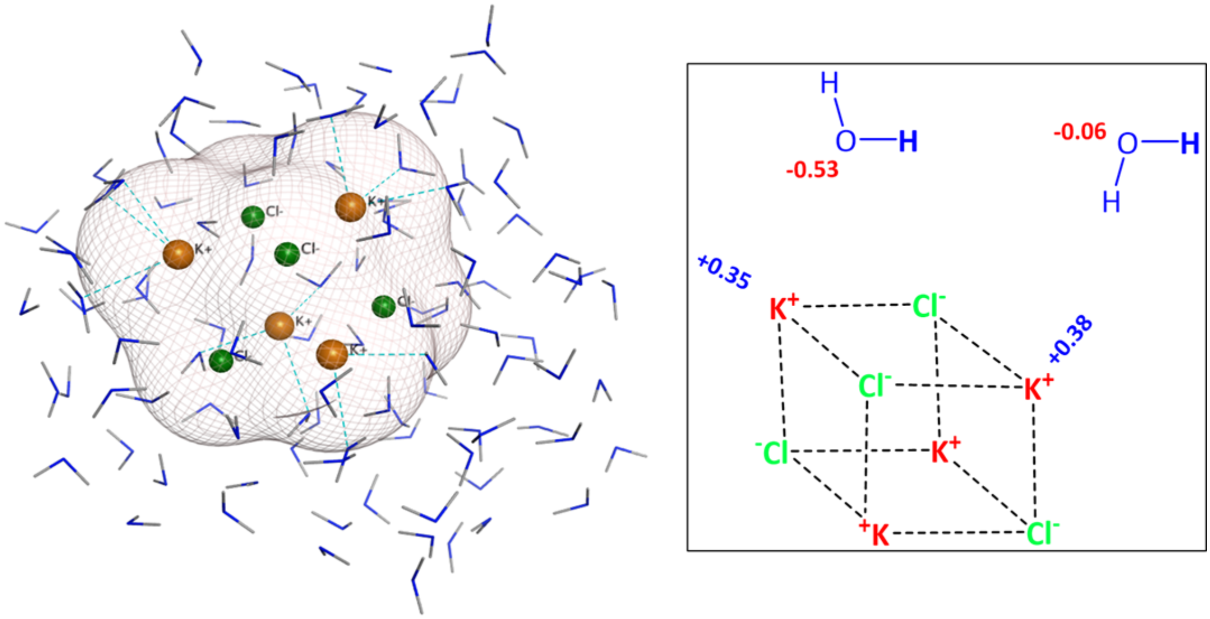

Figure S15. Optimized $(KCl)_4(H_2O)_{106}$ model 1 from Table S7. On the left the structure is reported, evidencing the ion positions, the water molecules around them and the van der waals surface of the $(KCl)_4$ unit The S_1 charge transfer characterized by HOMO/LUMO MO population is of 2O->2K type The computed S_1 charge transfer according to the Mulliken atomic population differences between S_1 and S_0 atomic charges computed at the charge S_0 minimum geometry (in electrons).

Tables S7. $(KCl)_4(H_2O)_{106}$ ions models. ΔE is the b-lyp/TZVP energy difference in kcal/mol; in the column type is reported the type of cluster obtained according to the ion positions among each other (see panel at the bottom of this table); CN K^+ and CN Cl^- are the number of K-O and Cl-H interaction respectively with threshold equal to 3Å; NH_b and <H_b> are the total number of H-bond interaction and the corresponding average O-H distance (in Å); 1ex is the band assignment according to the population of the HOMO->LUMO monoelectronic transition and $E(S_1)$ is the excitation energy in nm at the ground state geometry; S_1 opt kcl is the excitation energy (in nm) at the local minimum on the S_1 PEs upon TDDFT constrained geometry optimization; Δλ is the difference between emission and S_1 absorption excitation energy wavelengths (in nm).

	$(KCl)_4(H_2O)_{106}$	ΔE	CN K^+	CN Cl^-	CN KCl	KCl	NHb	<Hb>	E(S1)	1ex	S_1 opt kcl	Δλ
1	$(KCl)_4$	0,0	11	12	12	3,33	183	1,850	252,0	2O→2K	272,5	20,5
2	$(KCl)_4$	13,0	14	16	12	3,455	178	1,851	251,0	2O→3K	263,4	12,4
3	(KCl)(KCl) $2K^+$ $2Cl^-$	33,5	20	23	2	3,391	169	1,858	283,4	2O→2K,1H	315,7	32,3
4	$(KCl)_2(KCl)_2$	34,7	15	15	7	3,506	164	1,806	268,9	O→2K,2H	329,4	60,5
5	$(KCl)(KCl)_2$ (KCl)	36,1	13	15	8	3,413	169	1,817	298,9	2O→3K	318	19,1
6	$(KCl_3)(K_3Cl)$	36,7	13	14	7	3,333	169	1,821	277,3	2O→3K	300,0	22.7
7	$(KCl)(KCl)_2$ (KCl)	37,4	10	12	9	3,285	171	1,823	298,5	2O→3K	316,7	18,2
8	$(KCl)_3K^+Cl^-$	37,8	12	10	10	3,265	176	1,844	267,7	Cl→2K	647,2	379,5
9	$(KCl)_2(KCl)_2$	38,3	15	16	7	3,459	169	1,827	283,5	O→3K	298,7	15,2
10	$(KCl)_2(KCl)$	41,1	14	14	8	3,328	169	1,827	271,1	2O→K,2H	288,8	17,7

	$K^{+}Cl^{-}$											
11	$(KCl)_3K^{+}Cl^{-}$	44,3	15	16	9	3,407	168	1,838	271,2	2O→4K	299,7	28,5
12	$(KCl)_3Cl^{-}K^{+}$	52,3	15	17	8	3,522	168	1,842	287,6	O→2K,2H	307,5	19,9
13	$(KCl)_3K^{+}Cl^{-}$	60,3	15	18	6	3,286	167	1,848	296,8	O→2K,2H	401,1	104,3
14	$(KCl_3)(K_3Cl)$	60,6	10	11	10	3,253	177	1,858	290,8	2O→K,2H	585,5	294,7
15	$(KCl)_3Cl^{-}K^{+}$	70,3	15	17	7	3,478	166	1,837	269,0	2O→2K,2H	281,7	12,7
16	$(KCl)_3K^{+}Cl^{-}$	70,6	13	16	8	3,361	169	1,839	273,0	2O→3K,2H	287,3	14,3

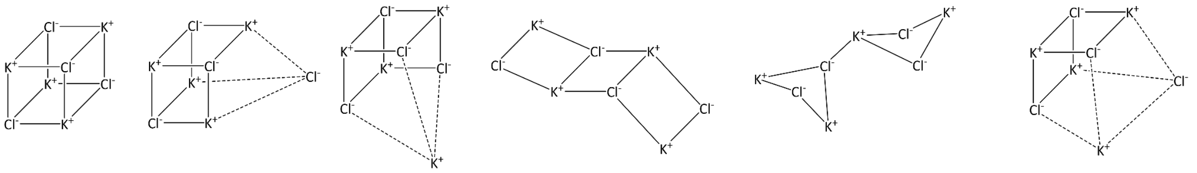

$(KCl)_4$ $(KCl)_3K^{+}\cdot Cl^{-}$ $(KCl)_3Cl^{-}\cdot K^{+}$ $(KCl)(KCl)_2(KCl)$ $(KCl)_2(KCl)_2$ $(KCl)(KCl)_2(K^{+}\cdot Cl^{-})$

Model 1 $(KCl)(H_2O)_{54}$ coordinates in atomic units.

164

Energy =

K	2.9596016	-3.6387789	0.7522546
Cl	1.1533324	-0.9832521	-1.8360939
O	4.0035296	-2.3379695	-1.6149052
H	4.7778956	-1.7335427	-1.4299311
H	3.2167750	-1.7583129	-1.7562380
O	2.6795963	-0.8625070	1.1907338
H	2.2322731	-0.6008876	0.3589681
H	2.1172905	-0.4695131	1.9313743
O	-1.0993654	-3.4377115	-1.6920912
H	-0.3492326	-2.8112520	-1.5719144
H	-0.9330508	-4.1880766	-1.0648564
O	2.5857475	-4.7128065	3.3955390
H	2.8277193	-5.6593816	3.5532130
H	3.0505329	-4.1572303	4.0669418
O	-1.2673896	0.4445836	-5.3479518
H	-2.0234129	0.2592613	-5.9320290
H	-0.8703700	1.3367893	-5.6625358
O	-0.7794718	-4.0914127	-4.4718090
H	-0.9781475	-3.9704629	-3.5093987
H	-0.3816025	-3.2266735	-4.7483779
O	0.2926032	-3.8109822	2.1203296
H	0.9107095	-4.2471307	2.7588153
H	-0.1465389	-4.5262674	1.6077550
O	5.3993567	-6.4301278	-6.1466893
H	4.4996617	-6.0251464	-6.3965340
H	5.6942216	-6.9082255	-6.9412140
O	4.1282686	-3.8034558	-3.8608744

H	3.6693347	-3.2862025	-4.5894790
H	4.1205091	-3.2280689	-3.0362726
O	6.7018524	2.2207998	-4.0367605
H	7.2561718	1.3658750	-4.1142777
H	7.3463498	2.9506249	-4.0387185
O	2.9683664	-2.5962223	-6.0117200
H	3.5438905	-1.8665481	-6.3885568
H	2.0867222	-2.1930288	-5.8023655
O	6.0628149	-8.1492409	-1.3833685
H	6.4264774	-9.0519837	-1.4002264
H	5.6256769	-8.0195468	-2.3018952
O	0.7985298	-6.0771400	-5.1792724
H	0.2378189	-6.8292625	-5.4359990
H	0.1598114	-5.3231776	-4.9153875
O	-0.4953039	-5.6084807	0.0485506
H	-1.0706444	-6.3924349	0.0563773
H	0.4331306	-5.9287309	-0.1806401
O	4.7173660	-0.7206500	-6.9434880
H	5.6192851	-1.0667755	-6.7482582
H	4.7064968	0.2065710	-6.6127677
O	6.5786494	-4.2016612	-4.9104283
H	6.3818469	-5.0176750	-5.4401703
H	5.6934667	-4.0215257	-4.4586626
O	7.2386844	-1.8980316	-6.1964065
H	7.0097700	-2.7820187	-5.7682969
H	7.8689154	-2.1035693	-6.9089043
O	-1.6224805	0.5298802	-2.5602003
H	-1.6121436	0.5295046	-3.5541926
H	-0.7523459	0.1413061	-2.3020324
O	3.0598924	-5.3147422	-6.8221400
H	3.0279613	-4.3306027	-6.7089997
H	2.2439956	-5.6374115	-6.3701655
O	2.3790001	2.9492495	-4.9675285
H	3.2057940	2.7079307	-5.4504873
H	2.4442110	2.4693509	-4.0957056
O	-0.9206736	-1.5075317	3.1949131
H	-0.6002453	-2.3617797	2.8095767
H	-1.6093691	-1.1660203	2.5756011
O	-2.8946820	-0.3664643	1.4372498
H	-3.7932563	-0.4375403	1.8045823
H	-2.9491887	-0.7869760	0.5173553
O	-1.0349822	5.2036761	-4.9892491
H	-1.1352705	5.9275861	-5.6319062
H	-0.1261632	5.3474998	-4.5345070
O	3.2121432	-7.4646984	3.3587192
H	3.5937647	-7.6111278	2.4558321
H	3.8050608	-7.9306377	3.9718674

O	2.7427181	-6.1013879	-3.2146923
H	1.9973543	-6.1838343	-3.8696752
H	3.2173316	-5.2526670	-3.4574290
O	8.3784068	-4.0995878	-2.7578123
H	8.0934445	-4.7708376	-2.0921726
H	7.7932083	-4.2279353	-3.5490650
O	-3.0687635	-1.3968741	-1.0302931
H	-2.5283654	-2.1992005	-1.2254258
H	-2.7269805	-0.7109416	-1.6549112
O	5.5309508	-4.9607327	0.9706190
H	5.8034446	-4.4007853	1.7628701
H	6.3281024	-5.0838854	0.4078669
O	0.2566533	2.6851699	2.7565909
H	-0.6033910	2.5650847	2.2210589
H	0.0033846	3.2039887	3.5400535
O	2.0605651	-6.0716133	-0.5582051
H	2.2754014	-6.1011324	-1.5337994
H	2.7229297	-6.6608285	-0.1115793
O	-1.9768563	2.3513915	1.3853581
H	-2.3188994	1.4237850	1.3728066
H	-1.9300691	2.6476982	0.4404775
O	5.3376047	1.4723717	3.1693505
H	6.0600081	1.9200340	3.6420134
H	5.0625662	2.1110809	2.4165847
O	6.0792885	-0.6726798	-0.9294321
H	6.0041036	-0.7173818	0.0710919
H	6.9808852	-0.9877243	-1.1975997
O	4.2278339	-7.3200997	0.7533343
H	4.7737668	-7.8107414	0.0950827
H	4.7442704	-6.4740691	0.9200724
O	2.5680401	1.8606747	-2.4360971
H	3.5077100	1.8234270	-2.0888147
H	2.1670778	0.9759221	-2.2637361
O	1.2230596	0.1080077	3.2390220
H	0.8977602	1.0401977	3.1176358
H	0.3976971	-0.4764174	3.2907513
O	3.5172436	-0.2765518	4.7468519
H	4.1246904	0.3560144	4.2996215
H	2.6278431	-0.1155144	4.3299640
O	1.0435246	3.9939064	-1.4098426
H	1.5633476	3.1969461	-1.6916440
H	0.0999806	3.6767987	-1.3472752
O	4.1240551	-2.8684988	4.9686094
H	4.2634164	-2.9912196	5.9235859
H	3.8891287	-1.8841826	4.8630834
O	2.2229708	4.1198372	1.1992166
H	1.5668785	3.6254966	1.7462144

H	1.7931933	4.2181551	0.3143637
O	-1.5999078	3.0504105	-1.3229043
H	-2.1535494	3.6935399	-1.8449384
H	-1.6650275	2.1852028	-1.8076329
O	-0.1717602	2.6963575	-6.1051476
H	-0.5858899	3.5359063	-5.7880908
H	0.7688007	2.7389818	-5.7873577
O	0.5496544	-1.6452986	-4.9693457
H	0.8166095	-1.4160320	-4.0458279
H	-0.0553162	-0.9047941	-5.2447007
O	5.1769129	1.8597739	-1.5799626
H	5.7091378	2.1434699	-2.3613643
H	5.5381297	0.9567986	-1.3570944
O	5.3935833	-0.7934634	1.6641696
H	5.5543845	0.0070360	2.2392158
H	4.4065811	-0.7684817	1.4965487
O	4.8668134	2.0797250	-6.0924906
H	5.5490569	2.1926678	-5.3686357
H	5.2074186	2.5722053	-6.8600613
O	4.8208062	-7.8561095	-3.6767423
H	4.0019984	-7.3131430	-3.5056914
H	5.1802986	-7.4761718	-4.5130711
O	4.7290904	3.0619107	1.1654126
H	3.8085885	3.4650583	1.1575897
H	4.9149627	2.7574001	0.2506233
O	8.1363113	0.0226835	-4.2921548
H	7.8494595	-0.6297201	-4.9779614
H	8.3192897	-0.5129452	-3.4794908
O	7.6373841	-5.9802228	-0.7453950
H	7.1392142	-6.8064037	-1.0141353
H	8.4191424	-6.2912902	-0.2560085
O	6.0923891	-3.2120465	2.9315644
H	5.9326587	-2.3285999	2.5094036
H	5.5193521	-3.1960120	3.7365609
O	8.5067281	-1.5780017	-1.9878317
H	9.3606656	-1.4666284	-1.5349726
H	8.4571690	-2.5620148	-2.2565638
O	1.3199006	5.3688900	-3.8412134
H	1.8591559	4.6771634	-4.2961712
H	1.2374449	5.0316220	-2.9065977
O	-2.9262593	4.8522593	-2.9613825
H	-3.1326669	5.7228970	-2.5807484
H	-2.3067762	5.0298821	-3.7187199

Model 1 $(KCl)_2(H_2O)_{108}$ coordinated in atomic units.
328
Energy =

K	-0.8947565	4.0869425	0.7719529
Cl	4.0817297	1.8173299	-0.2413172
K	1.9604990	-1.3291189	3.5811144
Cl	-1.3216052	-3.1824549	-3.6049520
O	8.1552140	0.2706919	1.4117734
H	7.2606642	-0.1305129	1.2469341
H	8.6874580	-0.4753605	1.7810156
O	4.3266892	0.7482284	-7.1538827
H	4.0124080	1.7508733	-7.1185171
H	4.7946541	0.6605473	-8.0030397
O	0.4563345	-0.3322064	-0.7692983
H	0.7092198	0.0823217	-1.6428657
H	0.6260746	-1.3030928	-0.8950005
O	-3.9362301	-1.3248448	-7.2640523
H	-3.7572816	-1.9693249	-7.9709662
H	-4.0584867	-1.8585219	-6.4146087
O	2.3234369	7.8843780	-2.7054446
H	1.4513191	7.7066785	-3.1284438
H	2.3137040	7.4283949	-1.8243177
O	-1.7156501	-3.2732589	1.9143318
H	-0.7687049	-3.4891154	2.1894671
H	-2.0078200	-2.5867425	2.5778006
O	6.3132191	6.0468820	-1.8449421
H	6.3319350	5.0847539	-2.0823187
H	5.7567693	6.4714650	-2.5462826
O	-4.1859755	-2.6162871	-4.9099585
H	-4.6578613	-3.4970751	-4.8694285
H	-3.2916243	-2.7638920	-4.4971921
O	-5.6299483	-4.3397431	3.0311626
H	-6.3952606	-4.8695986	3.3173187
H	-5.9409594	-3.8377766	2.2118024
O	4.0593292	-3.5736919	-6.7442121
H	3.8622185	-4.1166937	-5.9124892
H	4.0501094	-4.2008514	-7.4880600
O	-0.9027093	-6.2145512	-4.2149883
H	-1.2073258	-6.4042389	-5.1199181
H	-1.0485532	-5.2353550	-4.0813314
O	-8.5215733	0.3204438	-0.2434902
H	-8.4684905	-0.3887974	-0.9582200
H	-9.2369763	0.9206801	-0.5170161
O	-3.6915768	-5.4377388	-0.7219467
H	-3.3500265	-4.5029171	-0.8203958
H	-3.6107494	-5.6006014	0.2686363
O	-5.8702379	-5.9626712	-2.0962954
H	-5.9077912	-6.9362167	-2.1177419
H	-5.0450228	-5.7440953	-1.5280308
O	-6.5440361	-2.9488901	0.8990099

H	-5.9566620	-2.3338833	0.3674358
H	-7.0434887	-3.4757282	0.2258552
O	-2.4822347	2.5465645	-2.2073811
H	-2.6701610	2.1978015	-3.1203042
H	-3.3411276	2.9523268	-1.8957077
O	1.1541386	0.7391324	-3.2424758
H	0.7934203	0.2077961	-4.0070940
H	0.8208269	1.6741768	-3.3383111
O	-5.5951865	-4.9638427	-4.7239251
H	-5.6539796	-5.3355043	-3.8048474
H	-6.5130119	-4.9212568	-5.0431727
O	-1.6727483	1.2554670	0.0693139
H	-2.0613810	1.5664870	-0.7922259
H	-0.9994445	0.5762916	-0.1938948
O	-4.9627141	-1.2424429	-0.6076581
H	-4.0629768	-1.6673509	-0.6081030
H	-5.2020243	-1.1189309	-1.5667654
O	-2.6848420	-2.8574130	-0.6392040
H	-2.0624308	-2.7587641	-1.3968050
H	-2.1599061	-2.8987977	0.2119009
O	-5.9022395	1.2901795	0.2438980
H	-6.8544482	1.0680641	0.0726346
H	-5.4170079	0.4570690	0.0072784
O	-3.2293261	-5.5712628	1.9537713
H	-2.6433184	-4.7595861	2.0238525
H	-4.0411099	-5.3271519	2.4553897
O	-3.1938943	8.0387401	-0.4131010
H	-3.8937464	8.7065196	-0.3085907
H	-2.4985011	8.2538301	0.3070137
O	-8.3380868	-1.1640794	2.2621972
H	-8.5759453	-0.5493114	1.5316986
H	-7.7598474	-1.8259377	1.8141450
O	-7.8939962	-4.3449098	-1.1505962
H	-8.7082444	-4.8399185	-0.9535095
H	-7.2383678	-5.0056153	-1.5135224
O	4.9081716	-9.0311835	2.0875041
H	4.3250706	-8.7562005	2.8741594
H	4.8117466	-9.9950150	2.0131701
O	-0.0189254	2.3432654	3.0720319
H	0.5277549	1.7621101	2.4723036
H	-0.9329447	1.9553243	3.0750561
O	-5.0080620	4.0870132	4.2509560
H	-4.9501928	3.8396826	5.1895348
H	-5.1848072	3.2318077	3.7662941
O	4.1342938	-5.6961746	5.2815220
H	4.5287038	-5.6482229	6.1696537
H	4.7254574	-5.1412076	4.6833692

O	-6.6729973	-0.1051322	4.2403791
H	-7.3517421	-0.4566822	3.6014643
H	-6.1471867	-0.8847572	4.5250065
O	-2.4018222	-1.3969171	3.8413449
H	-3.2833822	-1.6284882	4.2235480
H	-2.4900473	-0.4972277	3.4031230
O	-0.3974429	-8.5634155	-0.2722398
H	-1.0596981	-8.1987443	-0.9423504
H	-0.4809983	-9.5320462	-0.3155181
O	-5.1864919	1.6960881	2.9237438
H	-5.7308309	1.0185389	3.4423497
H	-5.5218732	1.6345702	1.9904522
O	-2.8884417	5.1604555	2.8710173
H	-3.6050712	4.7627289	3.4399548
H	-2.6623089	6.0191381	3.3073339
O	0.3156665	-0.8960423	-5.2930131
H	-0.0140422	-1.7227970	-4.8699791
H	1.1111812	-1.1171920	-5.8894973
O	-0.1558914	7.5485512	-4.1973119
H	-0.1231663	8.3061932	-4.8078786
H	-1.0638377	7.6049441	-3.7565262
O	-5.7687221	-0.9208230	-3.2805632
H	-5.7654788	-0.0319311	-3.7355834
H	-5.2205389	-1.5124950	-3.8588245
O	-5.6448295	1.4827168	-4.7145470
H	-5.8773400	1.3435005	-5.6594042
H	-4.6526310	1.6561828	-4.7547394
O	-2.6208736	7.6729819	-3.1624174
H	-2.7779501	7.8104945	-2.1950021
H	-3.2997503	7.0373747	-3.4845301
O	-1.6966409	0.3653839	-6.5536366
H	-2.3444296	-0.3052298	-6.8680419
H	-0.9334132	-0.1379020	-6.1312475
O	-5.4754087	0.9615654	-7.6307811
H	-6.2571288	0.7335535	-8.1642886
H	-4.9480021	0.1173178	-7.5555526
O	-4.8547457	3.4862441	-1.1643782
H	-4.5019189	4.1973569	-0.5502062
H	-5.2545897	2.7613903	-0.6160250
O	-6.1502587	4.1147617	-3.5088009
H	-6.1232253	3.2451155	-3.9739901
H	-5.7894594	3.9234668	-2.5995053
O	0.4553574	6.4890895	1.5019900
H	1.2606075	6.5773254	0.9208058
H	0.7670371	6.0846780	2.3515230
O	2.3648727	-1.2880493	-6.9172807
H	2.9274736	-2.0980893	-6.8768195

H	2.9938141	-0.5263039	-7.0157820
O	-4.6356313	6.0297975	-4.5243349
H	-5.2422044	5.3087926	-4.1285083
H	-5.2307647	6.7035168	-4.8970682
O	-1.0069874	2.3015879	-8.5616410
H	-1.8987776	2.6991903	-8.6304916
H	-1.1297022	1.5673574	-7.9144979
O	4.1614184	4.2842346	-4.5819181
H	4.7592725	3.8666799	-3.9197581
H	4.2756877	5.2541395	-4.4523784
O	-8.2635209	-1.5667359	-2.1789154
H	-7.4264744	-1.3825940	-2.6792760
H	-8.2028493	-2.5075171	-1.9056512
O	-2.5165563	1.0496238	2.6528205
H	-2.3668250	1.0388610	1.6648779
H	-3.4599844	1.3552762	2.7916411
O	1.3900816	-5.0835044	5.0171457
H	1.0670089	-4.5884980	5.8197672
H	2.3584707	-5.2425591	5.1605371
O	1.6034400	0.6444128	1.5347552
H	1.2063107	0.2953323	0.6855396
H	2.4298616	1.0805151	1.2199216
O	5.6056159	0.6975622	5.0637884
H	5.3553814	-0.1180905	4.5635195
H	6.2218829	1.2171453	4.4668701
O	3.5731318	4.3819481	4.2821063
H	3.6783049	3.6336770	4.9209224
H	4.1645256	4.1825663	3.5112339
O	-0.0734832	6.7611457	5.5165609
H	0.3456308	6.0367524	4.9620861
H	0.6567740	7.3586121	5.7523758
O	4.6954447	-7.3750635	0.0185704
H	4.7748282	-8.0472619	0.7715938
H	3.7711513	-7.4678283	-0.3391539
O	5.9500562	3.3395768	-2.5023012
H	6.6339744	2.6818365	-2.7924573
H	5.4459996	2.9006538	-1.7791688
O	-1.3303973	-7.8446185	2.3985083
H	-1.0151665	-8.0227868	1.4831311
H	-2.0006669	-7.1317154	2.3227633
O	-3.5101924	5.3281423	0.2569073
H	-3.4459470	5.3521183	1.2567770
H	-3.5300525	6.2754676	-0.0310476
O	7.1942944	2.1605836	3.3729651
H	6.6676314	2.8329500	2.8823614
H	7.6385615	1.6043563	2.6876618
O	2.5084785	6.4011505	-0.3151074

H	2.2800060	5.4641511	-0.5683739
H	3.4462520	6.3862248	0.0144908
O	-1.4372521	8.4960638	1.5067519
H	-1.7842763	8.3839449	2.4220703
H	-0.6773253	7.8508594	1.4718426
O	-2.2026481	7.6191644	4.1358679
H	-2.8631946	7.9710610	4.7568940
H	-1.3947493	7.3775085	4.6911357
O	0.1108255	3.2998580	-3.1153642
H	-0.8161948	3.1912520	-2.7860978
H	0.0522745	3.9023833	-3.9150127
O	1.2590112	0.9508721	5.2473810
H	2.0609064	1.3930400	5.6367573
H	0.8346685	1.6063442	4.6456049
O	3.7769068	0.0533210	-2.9763507
H	2.8562997	0.3920034	-3.1847997
H	4.0084714	0.5278366	-2.1419056
O	2.0299609	-7.2411723	-0.8609849
H	1.9599762	-7.0915236	-1.8558215
H	1.2656860	-7.8244920	-0.6190133
O	6.0093163	-6.2414689	-4.2802369
H	6.2350351	-6.6274130	-3.4006624
H	6.7625949	-5.6381775	-4.4909519
O	4.5820339	-4.8059284	1.0498685
H	5.0104291	-4.1859418	0.3892732
H	4.7340263	-5.7374808	0.7218588
O	7.6878628	1.3403735	-3.4720921
H	8.4224820	1.6648268	-4.0223754
H	7.0548517	0.8749030	-4.0985551
O	6.8029692	-6.9688249	-1.6188830
H	7.4023532	-7.7282045	-1.5149821
H	6.0113246	-7.1674489	-1.0242789
O	9.7875779	-2.5860943	-0.5745949
H	9.1448156	-3.2287937	-0.9564756
H	9.5663612	-1.6987307	-0.9765513
O	8.2902143	-4.1751797	3.4192433
H	8.7445575	-4.9902376	3.1472400
H	8.7521645	-3.4369114	2.9330902
O	9.6161790	-2.1725248	2.0165086
H	10.5214545	-2.0148823	2.3380997
H	9.7072486	-2.3857114	1.0174718
O	3.7076564	-4.8507513	-4.4145884
H	4.5689313	-5.3652985	-4.2990247
H	3.7322014	-4.0850861	-3.7799379
O	9.1508910	-0.0050263	-1.2746760
H	8.5741239	0.3758191	-1.9728864
H	8.7969978	0.3053969	-0.4050760

O	5.5738244	-2.9898913	-0.7244626
H	4.8800584	-2.9151222	-1.4414943
H	6.4013424	-3.3466223	-1.1555893
O	7.9440095	-4.1884989	-4.5529504
H	7.4206845	-3.4684018	-5.0574176
H	8.8215812	-4.2270730	-4.9705566
O	5.2394325	3.8705093	2.0463384
H	5.3343254	4.7187577	1.5392298
H	4.8764085	3.2323829	1.3889833
O	5.5529589	-4.1735996	3.5534448
H	5.2370022	-4.4539961	2.6475003
H	6.5519147	-4.2047323	3.5212201
O	1.8420973	-6.7832014	-3.5120225
H	0.9384367	-6.5800388	-3.8421112
H	2.4459692	-6.0886909	-3.8805984
O	1.1156453	-2.9594362	-1.4082941
H	0.4413527	-3.2916659	-2.0431546
H	1.3299549	-3.6886931	-0.7464532
O	6.5526463	-2.4321951	-5.9095782
H	5.7221166	-2.8117253	-6.2867383
H	6.2921374	-1.5671231	-5.4936954
O	1.0431220	4.9589153	3.8039023
H	2.0243960	4.7394587	3.9707315
H	0.6027837	4.0892153	3.6594579
O	4.7087803	-1.4948458	3.4626676
H	5.0672243	-2.4112837	3.6042982
H	5.0403438	-1.2353602	2.5618496
O	1.9101910	-4.8173232	0.3834167
H	2.8760813	-4.7286383	0.6334743
H	1.8484643	-5.7342661	-0.0214471
O	3.4431106	-8.3121015	4.1678803
H	3.7570646	-7.4833595	4.5937060
H	2.4469745	-8.2321539	4.1461496
O	3.6573042	2.1255482	6.0124661
H	3.8752200	2.3028854	6.9441004
H	4.4304619	1.5434614	5.6493411
O	5.2001986	6.2605640	0.5203927
H	5.6800993	6.2028273	-0.3846144
H	5.5742330	7.0370330	0.9727994
O	5.6174001	-0.7733561	0.9208051
H	5.1172382	-0.0286530	0.5075185
H	5.6239898	-1.5268097	0.2659986
O	4.5656782	7.0315980	-3.8497135
H	4.8777887	7.7069731	-4.4758332
H	3.6919385	7.3795747	-3.4663654
O	-4.9004847	-2.3502568	4.8347131
H	-5.1547374	-3.1162687	4.2488481

H	-4.8666652	-2.7005214	5.7422267
O	-0.1950553	-1.3658680	5.5812909
H	0.1506126	-0.4445944	5.6894130
H	-1.0336310	-1.3266258	5.0540616
O	0.7346979	-7.8059098	4.3473849
H	0.0180744	-7.8437475	3.6656070
H	0.7743894	-6.8656314	4.6359557
O	0.8262131	-3.7678263	2.6689320
H	1.1992362	-4.2824262	1.9025102
H	0.9833576	-4.3170773	3.4903949
O	0.6020337	-3.4697759	7.0837462
H	0.0013366	-3.6857887	7.8156037
H	0.2306837	-2.6585142	6.6359794
O	-2.1654152	-7.4915065	-1.9940638
H	-1.7484289	-7.1091978	-2.8037635
H	-2.6411024	-6.7389047	-1.5641456
O	-0.2400722	4.9601282	-5.2758760
H	-1.1721461	4.8802787	-5.5990551
H	-0.1358291	5.9018034	-4.9775378
O	-3.0089244	2.0085604	-4.8448343
H	-2.4760526	1.3871654	-5.4270565
H	-2.9204578	2.9170516	-5.2501602
O	-3.8936700	3.2456022	-8.3992423
H	-4.2597453	3.6141449	-9.2215657
H	-4.4587690	2.4643543	-8.1704296
O	3.6722478	3.2096327	-7.0734486
H	2.7400579	3.4703170	-7.3239244
H	3.8608983	3.6249904	-6.1878722
O	1.1155093	4.0156173	-7.6653024
H	0.4242738	3.4164534	-8.0398520
H	0.7183475	4.3812003	-6.8419225
O	7.7783013	-4.2579901	-1.7801429
H	7.5535963	-5.2079664	-1.6221209
H	7.8865946	-4.1724580	-2.7661642
O	3.6018352	-2.6944454	-2.6093758
H	2.6985553	-2.7830966	-2.1961497
H	3.6821896	-1.7239483	-2.8268924
O	1.5428363	3.8546181	-0.8214500
H	2.2601853	3.1889270	-0.7067193
H	1.1675384	3.7031227	-1.7315090
O	5.7624693	0.0608048	-4.9244326
H	5.3278439	0.4075383	-5.7595720
H	5.0324523	0.0685176	-4.2465995
O	-2.9210001	4.4995980	-6.0318176
H	-3.5748217	5.1137208	-5.5957115
H	-3.2700469	4.2718863	-6.9253438

(1) Taniguchi, M.; Lindsey, J. S. Database of Absorption and Fluorescence Spectra of >300 Common Compounds for Use in PhotochemCAD. *Photochem. Photobiol.* **2018**, *94* (2), 290–327.
(2) Rhys Williams, A. T.; Winfield, S. A.; Miller, J. N. Relative Fluorescence Quantum Yields Using a Computer-Controlled Luminescence Spectrometer. *Analyst* **1983**, *108* (1290), 1067–1071.
(3) Chen, R. F. Fluorescence Quantum Yields of Tryptophan and Tyrosine. *Anal. Lett.* **1967**, *1* (1), 35–42.
(4) Lakowicz, J. R. Principles of Fluorescence Spectroscopy. 1999. https://doi.org/10.1007/978-1-4757-3061-6.
(5) Menter, J. M. Temperature Dependence of Collagen Fluorescence. *Photochem. Photobiol. Sci.* **2006**, *5* (4), 403–410.
(6) Gallo, P.; Corradini, D.; Rovere, M. Ion Hydration and Structural Properties of Water in Aqueous Solutions at Normal and Supercooled Conditions: A Test of the Structure Making and Breaking Concept. *Phys. Chem. Chem. Phys.* **2011**, *13* (44), 19814–19822.
(7) Grimme, S.; Antony, J.; Ehrlich, S.; Krieg, H. A Consistent and Accurate Ab Initio Parametrization of Density Functional Dispersion Correction (DFT-D) for the 94 Elements H-Pu. *J. Chem. Phys.* **2010**, *132* (15), 154104.
(8) Becke, A. D. Density-Functional Exchange-Energy Approximation with Correct Asymptotic Behavior. *Physical Review A*. 1988, pp 3098–3100. https://doi.org/10.1103/physreva.38.3098.
(9) Lee, C.; Yang, W.; Parr, R. G. Development of the Colle-Salvetti Correlation-Energy Formula into a Functional of the Electron Density. *Phys. Rev. B Condens. Matter* **1988**, *37* (2), 785–789.
(10) *Ab Initio Calculation of Vibrational Absorption and Circular Dichroism Spectra Using Density Functional Force Fields*; 1995.
(11) Perdew, J. P. Density-Functional Approximation for the Correlation Energy of the Inhomogeneous Electron Gas. *Physical Review B*. 1986, pp 8822–8824. https://doi.org/10.1103/physrevb.33.8822.
(12) Perdew, J. P.; Ernzerhof, M.; Burke, K. Rationale for Mixing Exact Exchange with Density Functional Approximations. *The Journal of Chemical Physics*. 1996, pp 9982–9985. https://doi.org/10.1063/1.472933.
(13) Perdew, J. P.; Burke, K.; Ernzerhof, M. Generalized Gradient Approximation Made Simple. *Phys. Rev. Lett.* **1996**, *77* (18), 3865–3868.
(14) Performance of the TPSS Functional on Predicting Core Level Binding Energies of Main Group Elements Containing Molecules: A Good Choice for Molecules Adsorbed on Metal Surfaces. https://doi.org/10.1021/acs.jctc.5b00998.s001.
(15) Tao, J.; Perdew, J. P.; Staroverov, V. N.; Scuseria, G. E. Climbing the Density Functional Ladder: Nonempirical Meta–Generalized Gradient Approximation Designed for Molecules and Solids. *Physical Review Letters*. 2003. https://doi.org/10.1103/physrevlett.91.146401.
(16) Tao, J.; Perdew, J. P.; Staroverov, V. N.; Scuseria, G. E. Climbing the Density Functional Ladder: Nonempirical Meta–Generalized Gradient Approximation Designed for Molecules and Solids. *Physical Review Letters*. 2003. https://doi.org/10.1103/physrevlett.91.146401.
(17) Staroverov, V. N.; Scuseria, G. E.; Tao, J.; Perdew, J. P. Comparative

Assessment of a New Nonempirical Density Functional: Molecules and Hydrogen-Bonded Complexes. *The Journal of Chemical Physics*. 2003, pp 12129–12137. https://doi.org/10.1063/1.1626543.

(18) Yanai, T.; Tew, D. P.; Handy, N. C. A New Hybrid Exchange–correlation Functional Using the Coulomb-Attenuating Method (CAM-B3LYP). *Chemical Physics Letters*. 2004, pp 51–57. https://doi.org/10.1016/j.cplett.2004.06.011.
